# Development of optimal steam explosion pretreatment and highly effective cell factory for bioconversion of grain vinegar residue to butanol

**DOI:** 10.1186/s13068-020-01751-7

**Published:** 2020-06-24

**Authors:** Menglei Xia, Mingmeng Peng, Danni Xue, Yang Cheng, Caixia Li, Di Wang, Kai Lu, Yu Zheng, Ting Xia, Jia song, Min Wang

**Affiliations:** 1 State Key Laboratory of Food Nutrition and Safety, Ministry of Education, Tianjin Engineering Research Center of Microbial Metabolism and Fermentation Process Control, College of Biotechnology, Tianjin University of Science and Technology, Tianjin, 300457 China; 2 Key Laboratory of Industrial Fermentation Microbiology, Ministry of Education, Tianjin Engineering Research Center of Microbial Metabolism and Fermentation Process Control, College of Biotechnology, Tianjin University of Science and Technology, Tianjin, 300457 China

**Keywords:** Industrial vinegar residue, Steam explosion, Bioconversion, Kinetic model, Working mechanism, Integration of ARTP and repetitive domestication

## Abstract

**Background:**

The industrial vinegar residue (VR) from solid-state fermentation, mainly cereals and their bran, will be a potential feedstock for future biofuels because of their low cost and easy availability. However, utilization of VR for butanol production has not been as much optimized as other sources of lignocellulose, which mainly stem from two key elements: (i) high biomass recalcitrance to enzymatic sugar release; (ii) lacking of suitable industrial biobutanol production strain. Though steam explosion has been proved effective for bio-refinery, few studies report SE for VR pretreatment. Much of the relevant knowledge remains unknown. Meanwhile, recent efforts on rational metabolic engineering approaches to increase butanol production in *Clostridium* strain are quite limited. In this study, we assessed the impact of SE pretreatment, enzymatic hydrolysis kinetics, overall sugar recovery and applied atmospheric and room temperature plasma (ARTP) mutant method for the *Clostridium* strain development to solve the long-standing problem.

**Results:**

SE pretreatment was first performed. At the optimal condition, 29.47% of glucan, 71.62% of xylan and 22.21% of arabinan were depolymerized and obtained in the water extraction. In the sequential enzymatic hydrolysis process, enzymatic hydrolysis rate was increased by 13-fold compared to the VR without pretreatment and 19.60 g glucose, 15.21 g xylose and 5.63 g arabinose can be obtained after the two-step treatment from 100 g VR. Porous properties analysis indicated that steam explosion can effectively generate holes with diameter within 10–20 nm. Statistical analysis proved that enzymatic hydrolysis rate of VR followed the Pseudop-second-order kinetics equation and the relationship between SE severity and enzymatic hydrolysis rate can be well revealed by Boltzmann model. Finally, a superior inhibitor-tolerant strain, *Clostridium acetobutylicum* Tust-001, was generated with ARTP treatment. The water extraction and enzymolysis liquid gathered were successfully fermented, resulting in butanol titer of 7.98 g/L and 12.59 g/L of ABE.

**Conclusions:**

SE proved to be quite effective for VR due to high fermentable sugar recovery and enzymatic hydrolysate fermentability. Inverse strategy employing ARTP and repetitive domestication for strain breeding is quite feasible, providing us with a new tool for solving the problem in the biofuel fields.

## Background

Vinegar residue waste (VR) is a main byproduct of the vinegar brewing industry by solid-state fermentation of cereals and their bran [1]. In China, one of the world’s leading solid-state fermented vinegar producers, every ton of vinegar produced will bring 600–700 kg vinegar residue [2] and 2 million tons of VR are produced annually. Different from fruit vinegar in Europe, Traditional Chinese vinegar is made from various sorts of cereals and their bran, such as sorghum, sticky rice, wheat, millet bran, and wheat bran through solid-state fermentation. Because of lacking effective utilization strategy, this lignocellulosic material has traditionally been used as feed for cattle and other ruminants. However, ruminant diets made from VR have many disadvantages [3]. Comparing with ruminant diets, VR serves as a potential ideal substrate to produce biofuels such as hydrogen, biogas, ethanol and butanol through fermentation processes because of high content of cellulose and hemicellulose, low cost, abundance and easy availability throughout the year.

To date, there is no report on the usage of VR for butanol production. The utilization of VR mainly stems from two key elements. The first challenge arising from the complex and heterogeneous cell wall architecture makes VR recalcitrant to cellulase-catalyzed hydrolysis and subsequent bioconversion. Therefore, the application of an appropriate pretreatment method becomes quite essential [4] and the efficient one must meet the following requirements: (i) good accessibility of the cellulose component to hydrolytic enzymes; (ii) little or no degradation of solubilized hemicellulose and cellulose; (iii) insignificant formation of byproducts inhibitory to the subsequent hydrolysis and fermentation process; and (iv) cost effectiveness. Nowadays, steam explosion, ammonia fiber explosion, acid, alkaline and hydrothermal are among the more studied and better performing pretreatments [5]. Among all the pretreatment methods, steam explosion pretreatment is one of the most potential methods and has being explored extensively for the pretreatment of cellulose [6], hemicellulose [7], and lignin [8] because of effectiveness and inexpensiveness [9].

The second challenge is the inhibitors after the pretreatment. The main problems with the pretreatments are the generation of degradation products [5], mainly including furfural, 5-hydroxymethyl furfural and so on. The presence of these compounds will inhibit cell growth, substrate utilization and product synthesis, thus greatly reducing the production efficiency of lignocellulosic butanol [10]. To date, few natural strains can utilize lignocellulosic hydrolysate to produce butanol as efficiently as utilizing traditional starchy substrate [11]. Nowadays, great progress has been made on overexpression, insertion, knockout, and knockdown of the key genes in the ABE fermentation pathway and other relative genes (such as genes coding for heat-shock proteins, operon, transcription, etc.) [12]. However, due to the physiological complexity of solventogenic clostridia, recent efforts on rational metabolic engineering approaches to increase inhibitor tolerance of *C. acetobutylicum* are quite limited [13]. Since multiple largely unknown parameters determine a particular phenotype, an inverse strategy to select a phenotype of interest can be useful. Thus, developing rapid and diverse microbial mutation tool is of importance for strain improvement [14]. Nowadays, a new mutagenesis method for microbial mutation breeding using the radio-frequency atmospheric pressure glow discharge (RF-APGD) plasma jets named ARTP has gained widely attention and made great progress. By change the DNA sequences significantly, and it has been considered as a powerful tool for the microbial mutagenesis with its outstanding features, such as the low and controllable gas temperatures, abundant chemically reactive species, rapid mutation, high operation flexibility, etc. [14]. Besides, adaptive evolution is a set of environmentally induced mutations that confers growth advantages to cells. An organism is subjected to serial or continuous cultivation for many generations to which it is not optimally adapted to select more fit genetic variants. The development on these methods shed light on strain development.

This study focused on the two key bottlenecks of VR for butanol bioconversion. First, steam explosion pretreatment was established for the extraction of hemicellulose biopolymers from VR. The effects of varying temperature and retention time on fermentable sugars’ extraction and enzymatic digestibility of cellulose were studied systematically. Morphological and porous properties of changes in VR structure were presented and the relationship between SE severity and enzymatic hydrolysis rate was illuminated. Second, a novel method integration of ARTP mutagenesis and repetitive domestication was applied for the generation of high furfural-tolerant microorganism. Finally, a highly effective conversion system of steam-exploded VR for butanol was established.

## Results and discussion

### Removement of VR recalcitrance by SE

#### Composition of untreated and pretreated VR

The water content of the fresh vinegar residue just from the factory was 70.65 ± 4.57% (w/w). The components of solid vinegar residue based on the absolute dry weight are shown in Table 1. The results showed that carbohydrates accounted for about half of the components of solid vinegar residue, mainly including 25.53 ± 3.76% glucan, 17.08 ± 1.78% xylan and 5.83 ± 0.11% arabinan. Among other components, lignin content was 24.48 ± 0.06%, crude fat content was 11.80 ± 0.0%, and ash content was 5.92 ± 0.03%. The composition of vinegar grains varies slightly depending on the production process, raw material yield and season, which was quite similar with Brewer’s spent grain [5]. Compared with ordinary corn stalks, vinegar grains contained higher lignin (about 17.5% straw) [15] and lower protein (9.3%). Since high lignin quality will affect the palatability of feed, vinegar residue is not an ideal raw material for animal feed [5]. Considering the high content of carbohydrates in vinegar residue, it would be more reasonable to pretreat these carbohydrates to convert them into fermentable sugars for the synthesis of high value-added products [16, 17].

**Table 1:** The composition of raw vinegar residue based on the absolute dry weight

The composition content (g/100 g of raw material)			
Glucan	25.53 ± 3.76	Ash	5.92 ± 0.03
Xylan	17.08 ± 1.78	Soluble protein	0.58 ± 0.01
Arabinan	5.83 ± 0.11	Crude fat	11.8 ± 0.01
Lignin	24.48 ± 0.06	Other^a^	8.98 ± 3.00

^a^Other mainly represents crude protein (except for soluble protein) and a small quantity of micro-element [16, 17]

#### Chemical composition of washing liquid from exploded VR

To realize the high-value utilization of vinegar residue, we introduced steam explosion pretreatment in this study, and investigated the pretreatment effect of SE under different steam explosion pressure and residence time. Then, the water-soluble products accumulated on surfaces of pretreated VR were removed with deionized water [18]. The rest of the solid was used for enzymatic degradation. The chemical composition of washing liquid from exploded VR is shown in Table 2.

**Table 2:** Chemical composition of washing liquid from exploded VR

Pretreatment conditions			Composition of the washing liquid (g/100 g feedstock)						Glucan conversion (%)	Xylan conversion (%)	Arabinan conversion (%)
Pressure (MPa)	Time (min)	Severity (Log(R_0_))	Glucose	Xylose	Arabinose	Furfural	HMF	Total monosaccharides			
1.5	1	2.87^a^	4.18 ± 0.59	7.80 ± 0.51	1.86 ± 0.12	Not detected	Not detected	13.84 ± 0.41	14.74 ± 2.10	40.19 ± 2.67	9.58 ± 0.63
1.5	2	3.17^a^	4.46 ± 0.63	10.51 ± 0.61	2.58 ± 0.15	0.012 ± 0.001	0.010 ± 0.001	17.55 ± 0.46	15.72 ± 2.24	54.1 ± 3.20	13.29 ± 0.79
1.5	3	3.34^a^	6.62 ± 0.95	12.39 ± 0.71	3.79 ± 0.22	0.015 ± 0.002	0.011 ± 0.001	22.8 ± 0.63	23.34 ± 3.38	63.38 ± 3.72	19.53 ± 1.15
1.5	4	3.47^a^	8.50 ± 1.21	14.19 ± 0.93	4.62 ± 0.30	0.021 ± 0.003	0.034 ± 0.002	27.31 ± 0.81	29.97 ± 4.31	72.65 ± 4.87	23.81 ± 1.57
2	1	3.32^a^	2.82 ± 0.40	8.37 ± 0.57	2.16 ± 0.15	0.024 ± 0.003	0.028 ± 0.004	13.35 ± 0.37	9.94 ± 1.42	43.13 ± 2.99	11.13 ± 0.79
2	2	3.62^a^	7.43 ± 1.07	13.45 ± 0.87	3.64 ± 0.24	0.054 ± 0.008	0.063 ± 0.005	24.52 ± 0.73	26.2 ± 3.81	69.05 ± 4.56	18.76 ± 1.26
2	3	3.79^a^	7.64 ± 1.09	14.10 ± 0.85	3.81 ± 0.23	0.062 ± 0.007	0.097 ± 0.007	25.55 ± 0.72	26.94 ± 3.88	72.65 ± 4.45	19.63 ± 1.20
2	4	3.92^a^	9.07 ± 1.30	15.06 ± 0.91	4.25 ± 0.26	0.180 ± 0.011	0.230 ± 0.016	28.38 ± 0.82	31.98 ± 4.63	77.29 ± 4.77	21.9 ± 1.36
2.5	1	3.59^a^	5.22 ± 0.75	12.23 ± 0.61	2.62 ± 0.13	0.120 ± 0.007	0.192 ± 0.021	20.07 ± 0.5	18.4 ± 2.67	62.86 ± 3.20	13.5 ± 0.68
2.5	2	3.89^a^	8.36 ± 1.19	13.90 ± 0.71	4.31 ± 0.22	0.180 ± 0.013	0.220 ± 0.020	26.57 ± 0.71	29.47 ± 4.24	71.62 ± 3.72	22.21 ± 1.15
2.5	3	4.07^a^	9.80 ± 1.40	14.62 ± 0.72	4.79 ± 0.24	0.320 ± 0.045	0.456 ± 0.036	29.21 ± 0.79	34.55 ± 4.99	75.23 ± 3.77	24.68 ± 1.26

^a^ Compared with the untreated VR group, the content of monosaccharide from all the pretreated groups increased significantly by *t* test (*p* < 0.05)

**Fig. 1:**
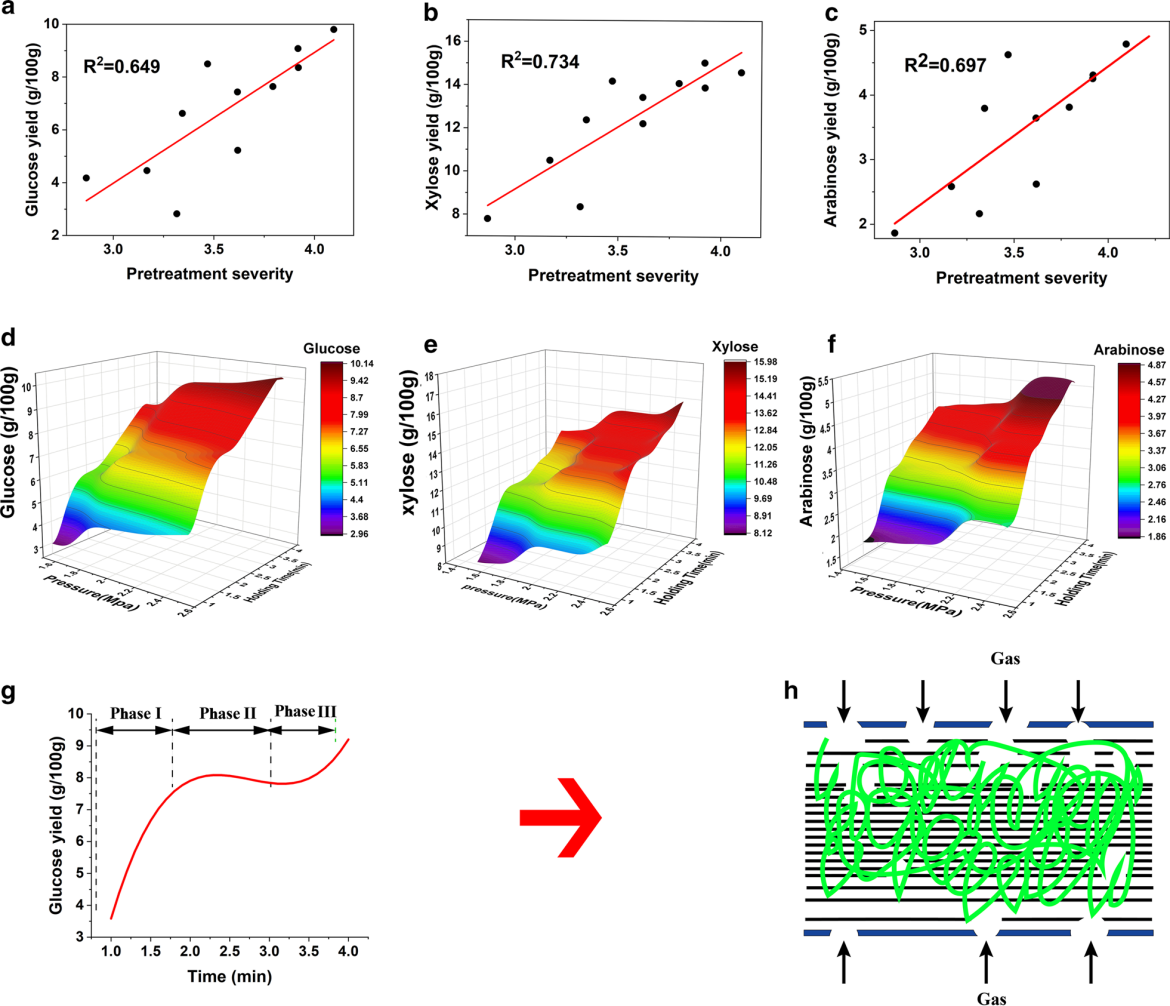
Fermentable sugars obtained in the water extraction after steam explosion pretreatment. **a**–**c** The correlationship between fermentable sugars in the water extraction and pretreatment severity of SE; **d**–**f** the fermentation sugars obtained under different operation conditions of SE (pressure and retention time); **f**–**h** the three phases of steam explosion

The result showed that the concentrations of fermentable sugars in the water extraction increased with pretreatment severity. The best results were achieved at the severity of 4.07 (holding pressure 2.5 MPa, residence time 3 min), with 9.80 ± 1.40 g glucose, 14.62 ± 0.72 g xylose and 4.79 ± 0.24 g arabinose per 100 g dry VR. The corresponding rate arrived at 34.55%, 75.23% and 24.68%. Obviously, the SE influences more obviously on hemicellulose depolymerization than that of cellulose [19, 20]. Figure 1a–c shows that the correlationship of SE severity with glucose, xylose and arabinose was 0.649, 0.734 and 0.697, respectively, indicating that there is not a simple linear relationship between the precipitation of monosaccharides and the SE severity. The result of two factors analysis of variance (ANOVA) indicated that the *p* values of pressure and holding time on the differences of the three sugars were glucose (0.04, 0.004), xylose (0.04, 0.006) and arabinose (0.03, 0.001), respectively, indicating that the holding time played a more significant role in the precipitation of monosaccharide than that of pressure.

To further represent the nonlinear relationship between independent and dependent variables, *Spearman correlation* analysis and the generalized additive model (GAM) were carried out. Detailed data analysis procedure was given in Additional file 1. In the *Spearman correlation* analysis, the correlation coefficient of *Spearman correlation* between operation parameter (severity, holding time, pressure) and glucose was 0.86, 0.82 and 0.47, respectively (Additional file 1: Table S1.1). According to the univariate GAM which employs single operation parameter as independent variable, the AIC (Akaike’s Information Criteria) values of pressure, holding time and severity were 63.6, 52.16 and 47.87, respectively. The Generalized Cross-Validation (GCV) and *R*^2^ (explained deviance) of the corresponding models were (6.23, 0.675), (10.63, 0.115) and (3.58, 0.78), respectively. The lower AIC and GCV represent higher interpretation degree [21–23]. Therefore, both models proved that the correlation between the operation parameters and glucose recovery followed the order: Severity > Holding time > Pressure.

To better understand the above nonlinear phenomenon, multivariable GAM was built based on multi-parameters. The best fitting model with the lowest GCV value and highest prediction accuracy was selected [23]. Result showed that the selected optimal multivariable GAM could effectively predict the variation of sugar recovery under different operation conditions. The detailed modeling process and prediction result are given in Additional file 1. Based on the GAM model, we introduced the 3D colormap surface to visualize all the experiment data, which took the pressure as the *x* axis, the holding time as the *y* axis, and the yield of simple sugar as the z axis. Figure 1d–f shows the data of glucose, xylose and arabinose obtained under different pretreatment conditions, respectively. Figure 1d–e provide a clearer angle of view to understand the in-depth rule. As a typical representative, Fig. 1g is a cross-section of Fig. 1d, showing the changes of glucose yield with holding pressure at 2 MPa with different hold time. We can more clearly find that the precipitation of the fermentable sugars can be divided into three phases (as shown in Fig. 1h): the stage of rapid increase, the stage of stagnation, and the stage of secondary increase. Similar phenomena have also been found in many other experiments [24]. The steam pretreatment can be divided into two stages: cooking phase and explosion phase. Based on our result, it is probable the working process of the cooking phase of steam explosion on the materials was a discontinuous process, which could include three steps listed as follows:

i) The lignin–carbohydrate complex linkage in the biomass cell wall structure was broken by the combined action of high-temperature water molecules and residual acetic acid, causing part of the lignin fragmentation and slight delignification. Meanwhile, cellulose and hemicellulose were exposed [25]. Some hemicellulose was hydrolyzed into low molecular weight xylan, xylose, arabinose, etc. Cellulose, mainly from the amorphous regions, hydrolyzed into lower molecular weight glucan and glucose [26]. Therefore, the fermentable monosaccharide content in the washing liquid increased continuously.
ii) As the holding time went on, hydrothermal pretreatment led to a complex series of reactions including rearrangements of lignin, the formation of pseudo-lignin, β-O-4 cleavage, changes in cellulose degree of polymerization and crystallinity, as well as cell wall porosity [27]. During this phase, energy was mainly used for structural alterations but not much monosaccharide was released.
iii) When reaching a significant intensity, the hydrothermal pretreatment disrupted the biomass cell wall matrix and facilitated the hemicellulose degradation. Especially, cellulose crystallinity got effectively decreased [28, 29]. Therefore, the fermentable sugar content in the reaction medium increased again.

Temperatures > 180 °C led to parallel reactions of solubilization of hemicellulose and lignin compounds, resulting in an extract enriched with phenolic and heterocyclic lignin composites; the product pattern of hemicellulose degradation also shifted to the formation of furfural and hydroxymethyl-furfural (HMF) [30]. All these compounds tend to inhibit biological processes like fermentation including biobutanol production. It should be noted that the concentrations of furfural and HMF increased sharply when the SE severity was higher than 3.90. Therefore, the optimum operation condition was 2.5 MPa pressure and 3 min residue time considering higher sugar yield and less inhibitors generated.

#### Enzymatic hydrolysis of SE-VR

The essence of lignocellulose pretreatment is the acquisition of fermentable monosaccharides, especially glucose, which can be realized through enzymatic hydrolysis. Therefore, enzymatic efficiency lies on the core of the conversion process. To determine the effect of SE on the enzymatic properties of VR, steam explosion-pretreated vinegar residue (SE-VR) was enzymatically hydrolyzed with solid loadings at 5% (w/w). Non-pretreated VR was used as a control. The enzymatic hydrolysis result are shown in Table 3, respectively.

**Table 3:** The enzymolysis result of SE-treated VR and untreated

Pretreatment conditions			Composition of SE-treated VR (%)			Enzymolysis result^a^					
Pressure (MPa)	Time (min)	Severity (Log(R_0_))	Glucan	Xylan	Arabinan	Glucose (g/100 g)	Xylose (g/100 g)	Arabinose	Glucose recovery (%)	Xylose recovery (%)	Arabinose recovery (%)
								(g/100 g)			
1.5	1	2.87	27.61 ± 3.59	6.59 ± 0.99	2.77 ± 0.22	8.96 ± 0.90	3.03 ± 0.33	2.15 ± 0.15	29.14 ± 3.26	40.47 ± 5.01	68.31 ± 5.42
1.5	2	3.17	25.86 ± 3.36	6.43 ± 0.77	2.63 ± 0.13	10.02 ± 0.90	3.06 ± 0.21	2.39 ± 0.31	34.88 ± 3.48	41.88 ± 3.27	79.98 ± 11.79
1.5	3	3.34	24.37 ± 2.19	5.19 ± 0.26	2.09 ± 0.13	11.78 ± 1.41	3.69 ± 0.48	2.66 ± 0.19	43.51 ± 5.79	62.57 ± 9.25	112.01 ± 9.09
1.5	4	3.47	23.38 ± 2.57	3.72 ± 0.52	1.76 ± 0.25	13.16 ± 1.58	4.01 ± 0.28	2.74 ± 0.41	50.66 ± 6.76	94.87 ± 7.53	137.02 ± 23.30
2	1	3.32	24.58 ± 1.23	6.44 ± 0.97	2.20 ± 0.26	11.59 ± 1.51	3.36 ± 0.34	2.48 ± 0.20	42.44 ± 6.14	45.92 ± 5.28	99.21 ± 9.09
2	2	3.62	22.05 ± 1.10	4.18 ± 0.50	1.88 ± 0.15	14.66 ± 1.17	2.41 ± 0.29	1.94 ± 0.14	59.84 ± 5.31	50.74 ± 6.94	90.82 ± 7.45
2	3	3.79	21.87 ± 2.19	3.91 ± 0.51	1.56 ± 0.23	16.05 ± 1.93	1.89 ± 0.26	1.98 ± 0.14	66.05 ± 8.82	42.54 ± 6.65	111.71 ± 8.97
2	4	3.92	21.76 ± 2.83	3.81 ± 0.50	1.24 ± 0.06	17.06 ± 2.05	1.99 ± 0.30	2.00 ± 0.22	70.57 ± 9.42	45.97 ± 7.87	141.95 ± 17.74
2.5	1	3.59	22.05 ± 3.31	6.05 ± 0.54	2.08 ± 0.19	15.07 ± 0.90	2.85 ± 0.31	2.21 ± 0.22	61.52 ± 4.08	41.46 ± 5.12	93.51 ± 10.58
2.5	2	3.89	21.76 ± 1.31	3.90 ± 0.47	1.29 ± 0.12	19.92 ± 1.20	1.78 ± 0.11	2.16 ± 0.17	82.4 ± 5.51	40.17 ± 2.82	147.37 ± 13.18
2.5	3	4.07	21.71 ± 2.39	3.52 ± 0.21	1.19 ± 0.16	20.18 ± 1.62	2.30 ± 0.14	2.01 ± 0.28	83.66 ± 7.46	57.51 ± 3.98	148.66 ± 23.53
Untreated			25.53 ± 3.83	17.08 ± 2.56	5.83 ± 0.70	7.05 ± 0.92	2.95 ± 0.44	2.25 ± 0.16	24.86 ± 3.60	14.59 ± 2.58	33.97 ± 2.74

^a^ The enzymatic hydrolysis conditions were pH 5.0, 150 rpm, 50 °C, 72 h, a solid load of 5% (w/w) and the enzyme load was adjusted to 15 FPU/g DM of Celluclast 1.5 L and 15 U/g DM of β-glucosidases

In Table 3, it can be found that glucose obtained quickly increases with the SE severity from 7.05 ± 0.92 g/100 g to 20.18 ± 1.62 g/100 g, which almost increased by 2.86-fold. The best result achieved with the glucose recovery rate reached as high as 83.66% when the pressure and residence time were set at 2.5 MPa and 3 min, respectively. The concentrations of xylose and arabinose ranged from 1.78 ± 0.11 g/100 g to 4.01 ± 0.28 g/100 g. Obviously, glucose serves as the main product which is rather higher than xylose and arabinose. It mainly arises from the reason that the SE pretreatment is more effective on the depolymerization of hemicellulose, and thus most xylose and arabinose were solubilized in the pretreatment liquid. Meanwhile, an arabinose recovery rate higher that 100% was noted. This may be related to the composition of commercial cellulase. Although cellulases are enzymes involved in the cellulose hydrolysis, the commercial cocktails also contain several accessory enzymes, such as xylanase and xylosidase, which are able to attack hemicellulose, releasing xylose and arabinose [31].

**Fig. 2:**
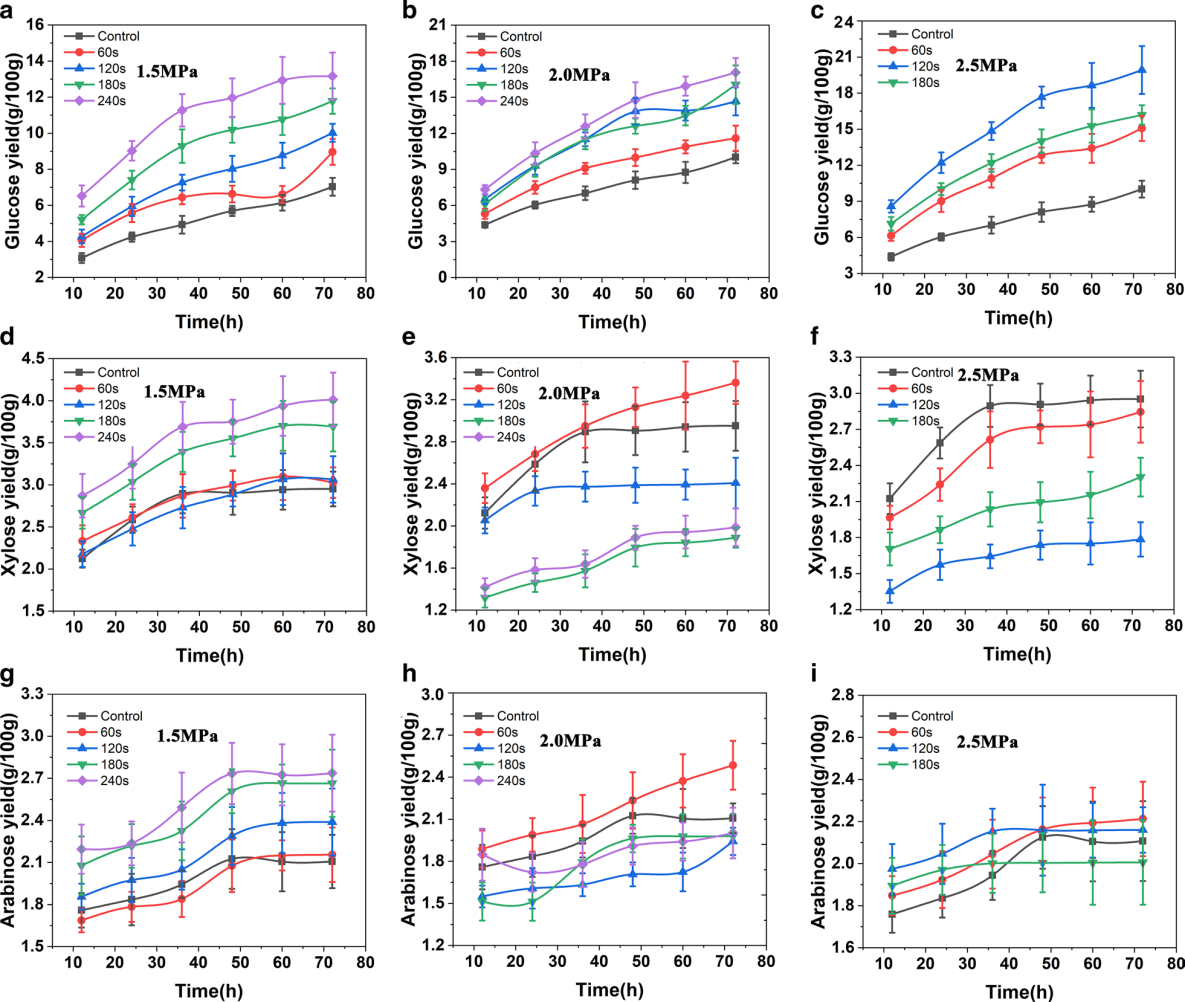
The enzymatic hydrolysis process of vinegar untreated and pretreated with steam explosion. **a**–**c** The glucose yields from the hydrolysis of VR with different pretreatment conditions at different enzymolysis time. **d**–**f** The xylose yields and **g**–**i** the arabinose yields, respectively. The *X*-axis represents the enzymolysis time. The SE pressures are 1.5, 2.0 and 2.5 MPa, which are noted on the figures. The VR without SE pretreatment was set as control

**Fig. 3:**
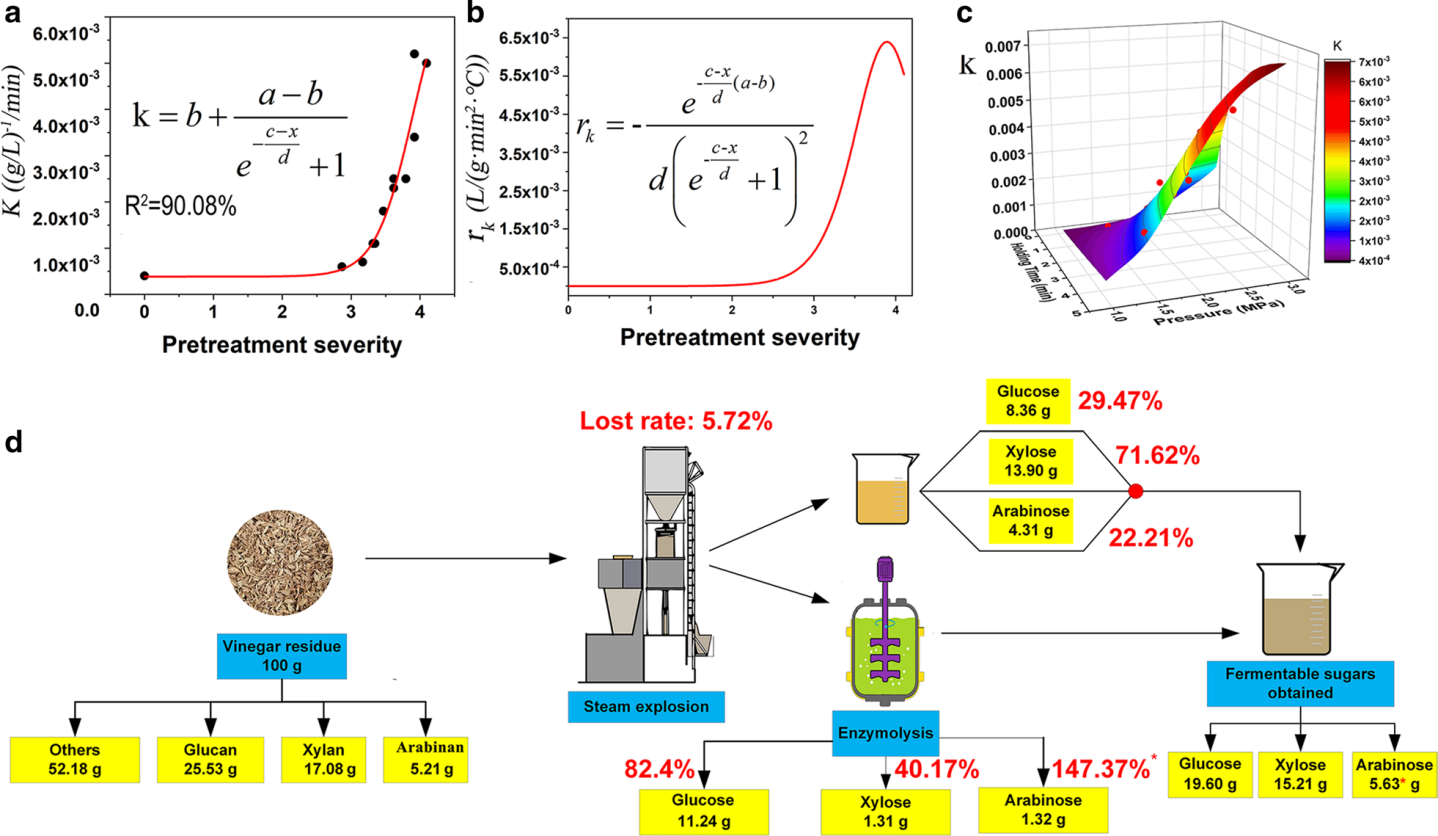
The relationship between steam explosion and enzymatic hydrolysis. **a** The relationship between the enzymatic hydrolysis rate constant *k* and pretreatment severity; **b** the change rate of the enzymatic hydrolysis rate constant *k* under different pretreatment severity; **c** the agreement between the mathematical model and the actual experiment data; **d** the mass balance of the steam explosion treatment in the optimal condition

The detailed enzymatic hydrolysis is shown in Fig. 2. To understand the process more rationally, the kinetic behavior of enzymatic hydrolysis with different SE-VR was studied using empirical statistical modeling method, which was developed from the work of Ding et. al [32]. The detailed model derivation process is given in Additional file 2. The rate equation for the enzymatic hydrolysis is expressed as Eq. 1.

1 $$\frac{\text{dc}}{dt} = - kc^{2}$$

where c is the monosaccharide concentration in the enzymatic hydrolysate (g/L), t is the enzyme time (*h*) and *k* represents the enzymatic hydrolysis rate constant (g/L)^−1^/h. The higher *k* represents the higher enzymatic hydrolysis rate. The *k* values of glucose, xylose and arabinose from different hydrolyzing experiments are given in Table 4. It can be concluded that the SE can effectively improve the hydrolysis efficiency of VR. The *k* value of the untreated VR was only as low as 0.0004 (g/L)^−1^/h. With the increase of SE severity, the k value increases quickly. The highest k value, 0.0051, came from the group where the SE operation condition was 2.5 MPa, 2 min, and was as high as 13-fold of that from the control group. Through data fitting, it was found that this correlation can be well described by Boltzmann (as shown in Eq. 2) with an *R*^2^ as high as 90.08%, indicating that there exists a stable correlation between *k* and SE severity (Shown in Fig. 3a). The concrete process of the model fitting and the result evaluation for Eq. 2 are shown in Additional file 3.

2 $$y = \frac{{{\text{a}} - b}}{{1 + e^{(x - c)/d} }} + b$$

where *a*, *b*, *c* and *d* are constants, *y* is the value of *k* and *x* is the SE severity.

**Table 4:** The kinetic parameter *k* of the kinetic model of enzymatic hydrolysis reaction

Pretreatment	Glucan	Xylan	Arabinan
1.5 mPa, 1 min	0.0006	0.0052	0.0446
1.5 mPa, 2 min	0.0007	0.0053	0.0870
1.5 mPa, 3 min	0.0011	0.0177	0.2214
1.5 mPa, 4 min	0.0018	0.1443	0.0633
2.0 mPa, 1 min	0.0011	0.0061	0.1824
2.0 mPa, 2 min	0.0025	0.0147	0.2182
2.0 mPa, 3 min	0.0025	0.0085	0.2667
2.0 mPa, 4 min	0.0034	0.0103	0.5035
2.5 mPa, 1 min	0.0023	0.0054	0.6214
2.5 mPa, 2 min	0.0052	0.0084	0.9981
2.5 mPa, 3 min	0.0051	0.0190	1.9238
Control/1	0.0004	0.0004	0.0038

The change rate of *k* value can be obtained by deriving Eq. 2 with *x* (SE severity). Then, a deep insight into the effect of SE on the enzymatic hydrolysis was achieved which is shown in Fig. 3b. Similar to our hypothesis on the cooking process, the effect of SE on enzymatic hydrolysis rate also can be divided into three phases: in Phase I where SE severity within the range of 0–2, the enzymatic hydrolysis rate (*k*) increases slowly; in Phase II where SE severity within the range of 2–3.8, the *k* value grows exponentially; when SE severity higher than 3.8, the *k* goes down fast again. Figure 3c shows the distribution of experiment data and our proposed model. The result of experiment data and simulation result proved that the accuracy and reliability of our proposed models (*R*^2^ = 91.00%).

Mass balance of the steam explosion process is shown in Fig. 3d. Taking the optimal operation condition (pressure 2.5 MPa, hold time 2 min) as the final solution, 100 g VR extract 8.36 g glucose, 13.90 g xylose and 4.31 g arabinose can be obtained in the water extraction. After enzymatic degradation, the solid residue can provide 11.24 g glucose, 1.31 g xylose and 1.32 g arabinose. Finally, a total of 19.60 g glucose, 15.21 g xylose and 5.63 g arabinose can be obtained.

#### Morphological and porous properties of SE-treated sample

The accessible surface area within the substrate is a key factor for the saccharification of plant cell walls by cellulolytic enzymes. In most cases, pretreatment enhanced cellulose hydrolysis by enlarging accessible and susceptible surface area. To reveal the underlined working mechanisms of SE, morphological and porous properties of treated substrate were analyzed.

**Fig. 4:**
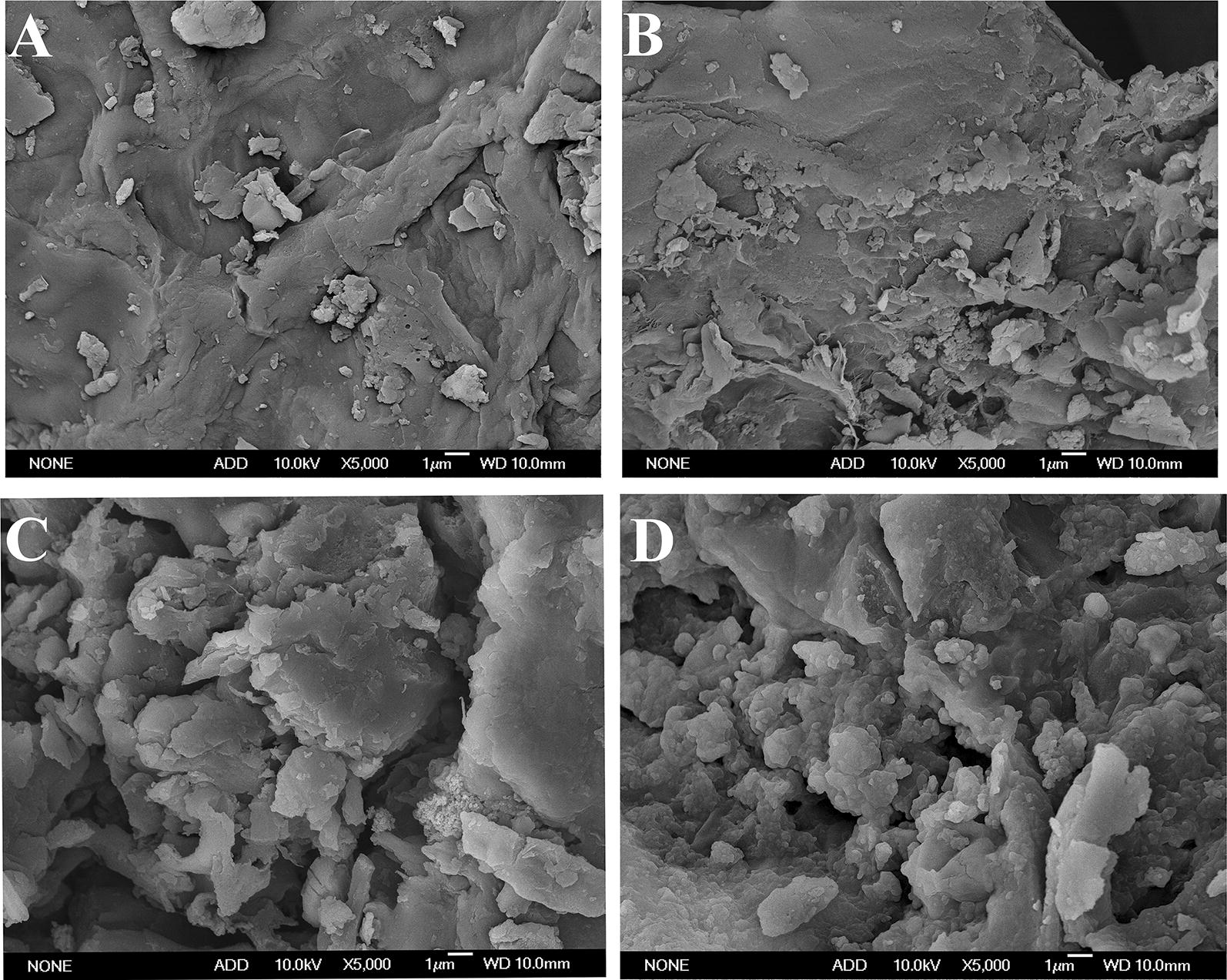
The ultrastructure images of vinegar residue before and after steam explosion treatment. **a** The control (untreated vinegar residue); **b**–**d** the treated VR from 1.5 MPa to 2 min, 2 MPa–2 min and 2.5 MPa–2 min respectively

Figure 4a–d shows a large variation of the VR ultrastructure before and after steam explosion pretreatment. As representatives, Fig. 4b–d shows the images of VR from 1.5 MPa–2 min, 2 MPa–2 min and 2.5 MPa–2 min operation conditions, respectively, with Fig. 4a as a control. Based on our result, SE rendered the morphology of the VR disorganized, rugged and rough and led to an overall reduced practical size, which became more evident with the increase of pretreatment severity. This phenomenon might be mainly caused by the coupling effect of the hydrolytic chemical reactions and intense shearing forces of SE [33]. During the SE process, characteristic species got dissolved, such as, the releasing of silica, breaking of intra-molecular H-bonding of microfibril, liberation of amorphous pectin, hemicellulose, lignin and so on [34]. Therefore, the fibrous strands got disentangled and loosened and the fibrous network trended to be depolymerized progressively [33]. The hemicellulose is largely removed thus leaving holes in the substrate. This kind of polyporus and covered with exposed cellulose morphology will be quite suitable for the full contact between substrate and enzyme molecules, contributing to the increase of hydrolysis efficiency [34].

**Fig. 5:**
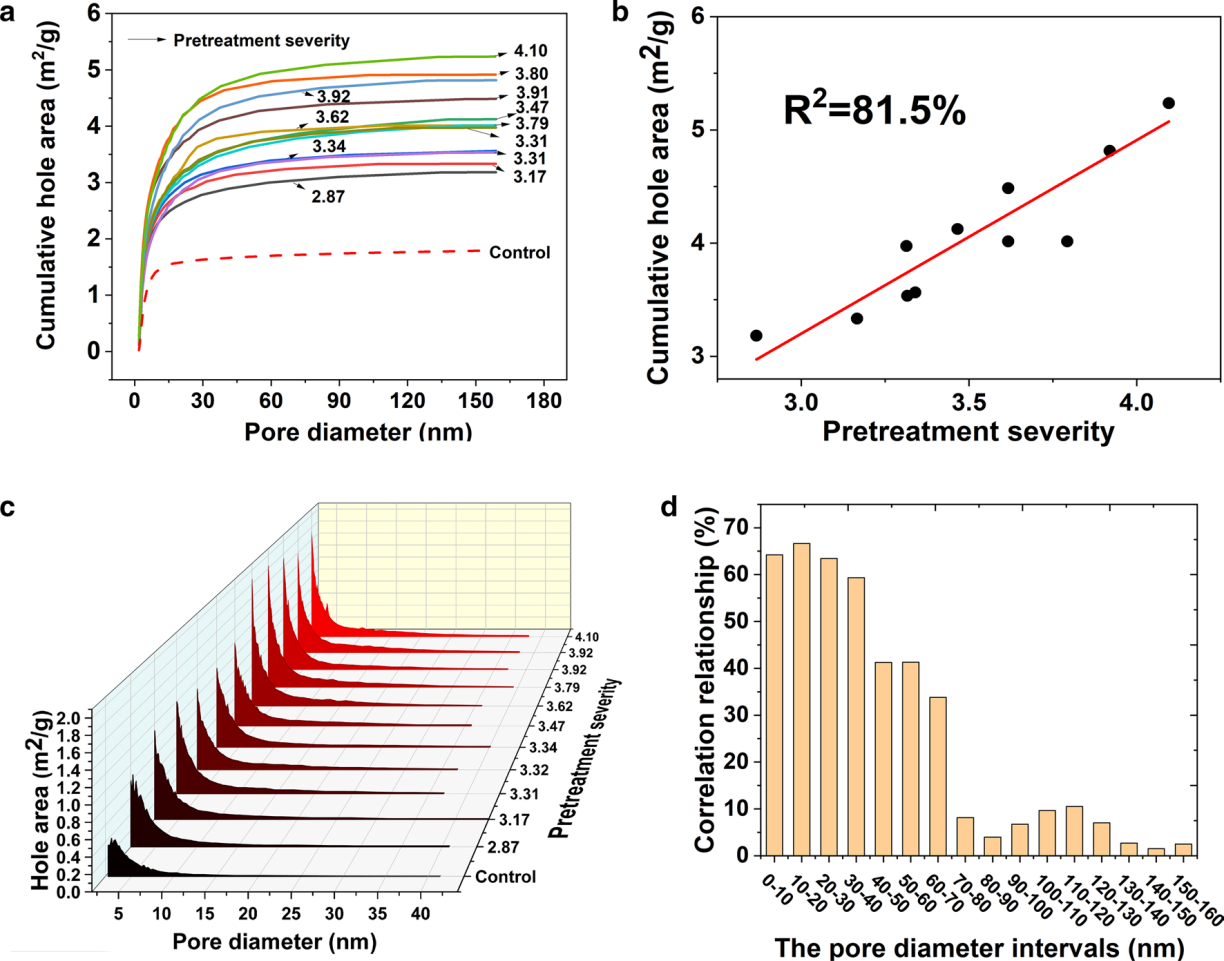
The changes of cumulative hole area, hole area and pore diameter under different pretreatment severity. **a** The relationship cumulative hole distribution of VR under different pretreatment severity; **b** the correlationship between the cumulative hole area and pretreatment severity; **c** the hole area distribution; **d** the correlationship between pretreatment severity and hole area within different pore diameter intervals

Obviously, Fig. 4 cannot be used as evidence that increasing severity of SE pretreatment decreased particle size. To better understand the significant influence of SE on the porous structure of VR, the pore distribution characteristics of pretreated VR were investigated by the N2 adsorption method. Figure 5a shows the cumulative hole area changes with pore diameter under different treatment conditions. We can find that the pores with diameter within 30 nm were quickly increased after SE treatment. The correlation analysis shows that an obvious linear relationship exists between the pretreatment severity and cumulative hole area with the *R*^2^ at 81.5% (Fig. 5b), suggesting that the more severe the SE employed, the more the hole area becomes. Comparing with the control group, the cumulative hole area from 2.5 MPa–3 min increased by 2.2-fold. Meijuan et al. [35] and Stone et al. [36] proved that initial rate of hydrolysis is a function of cellulose’s accessible surface area. Ladisch et al. [37] studied the leveling off of the cellulose particle size during cellulase treatment. They hypothesized that enzymatic hydrolysis of the microcrystalline cellulose was dominated by a tunneling mechanism—the enzyme complex attacked the cellulose by penetrating into the interior of the particle rather than eroding the outer surface. These previous researches explained quite well our results on the enzymatic hydrolysis rate variations between different treatment conditions. Furthermore, we analyzed the pore distributions under different pretreatment conditions, for which data are given in Additional file 4. Figure 5c only shows the hole area within the pore diameter distribution within 50 nm. Figure 5d shows the correlation between the cumulative hole area from different pore diameter intervals and pretreatment severity. We found that the steam explosion mainly generated the holes with diameter within 10–20 nm, followed by 0–10, 20–30, 30–40, and 40–70 nm. Chesson et al. founded that the wall matrix of wheat straw was disrupted by acid erosion to produce a significant pore distribution seen in 3–20 nm [38]. Zhao et al. [39] studied the correlation of porous structure, mass transfer and enzymatic hydrolysis of steam-exploded corn stover, finding that steam explosion mainly increased the pores with 5–9 nm diameter. Those differences mainly arise from the variations on substrate characteristics and operation method and conditions. It was important that pore size > 3 nm had an essential accessibility effect of enzyme protein molecule into plant active cell site [40]. Pore existence in biomass materials was also an evidence of some polymer components’ dissolution and leaving available constituents such as cellulose, lignin in residue that was further utilized as biofuel [34]. We discovered that steam explosion effectively generates holes with diameter within 10–20 nm, which facilitated the mass transfer process and enhance the hydrolysis efficiency.

### Generation of inhibitor-tolerant strain and biobutanol fermentation

**Fig. 6:**
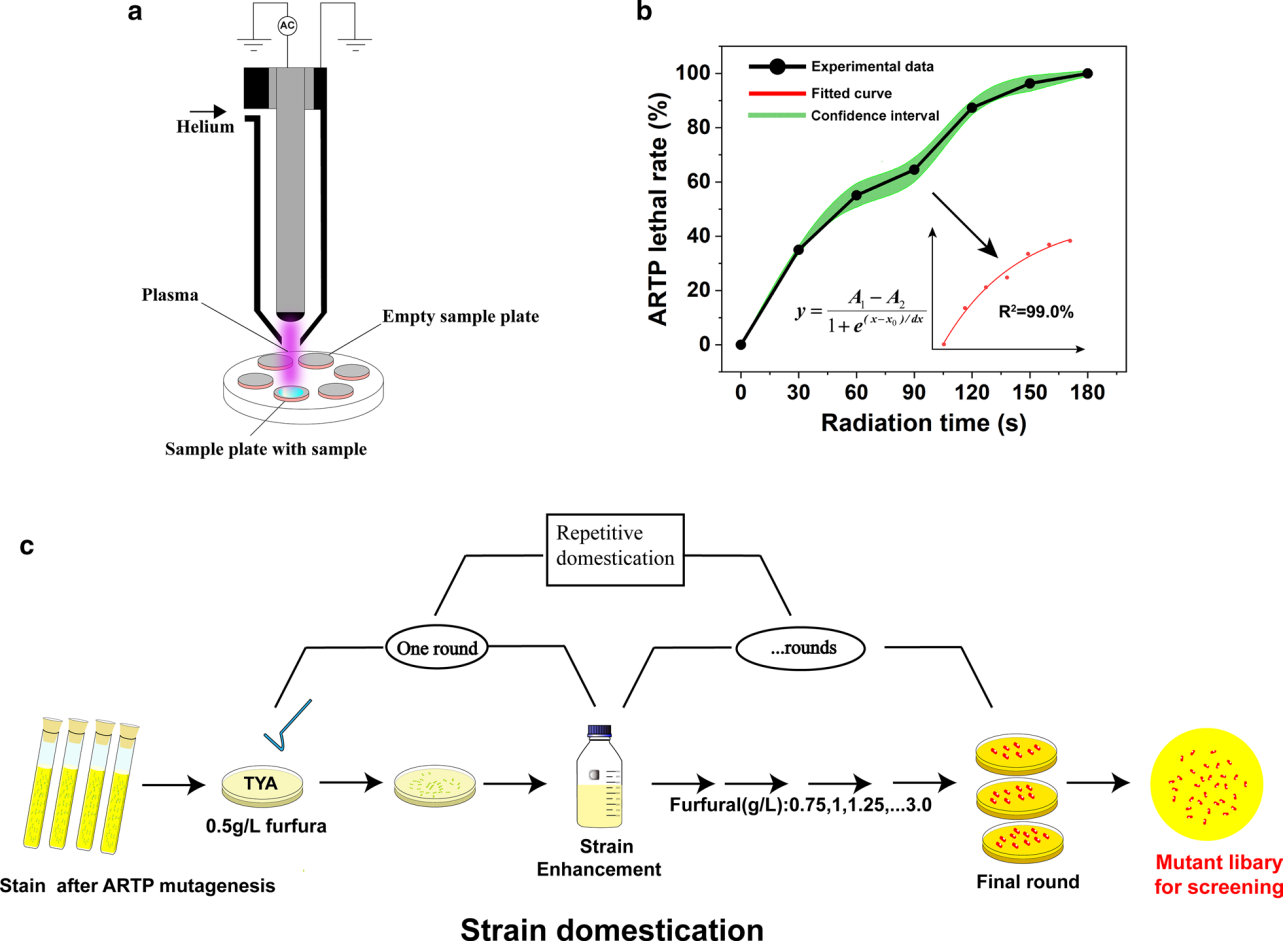
*C. acetobutylicum* Tust-001 obtained from ARTP mutagenesis. **a** The schematic diagram of the ARTP equipment; **b** effects of different plasma treatment times on the survival rate of *C. acetobutylicum* ATCC 824; **c** the screening process of *C. acetobutylicum* Tust-001

Figure 6a shows the schematic diagram of the ARTP equipment [41]. Figure 6b shows effects of different plasma treatment times on the survival rate of *C. acetobutylicum* ATCC 824. Figure 6c shows the workflow of repetitive domestication. According to previous reports, a survival rate of 10% is considered appropriate; thus, 150 s was chosen as optimal for mutation. After treatment with plasma radiation, the strain was spread and cultivated on plates for 48 h at 37 °C in the anaerobic incubator. After about 5 rounds of domestication, more than 110 mutant colonies were harvested on the plates containing 3 g/L furfural. They were further inoculated into the VR pretreated solvent for fermentation performance test.

As a control, *C. acetobutylicum* ATCC 824 grew slowly (OD_600_ at 60 h was only 1.32 ± 0.14) and a typical “acid crash” phenomenon was observed [42], where only about 3.42 ± 0.26 g/L acetic acid and 2.56 ± 0.18 g/L butyric acid were produced (Data not shown). Though previous study showed that inhibitor effect occurred only when the concentration of HMF or furfural was greater than 0.5 g/L [43–45]. Strong synergistic effect among the inhibitors produced by SE pretreatment was observed in our study. Similar results also have been observed in some other literatures [46–49].

**Fig. 7:**
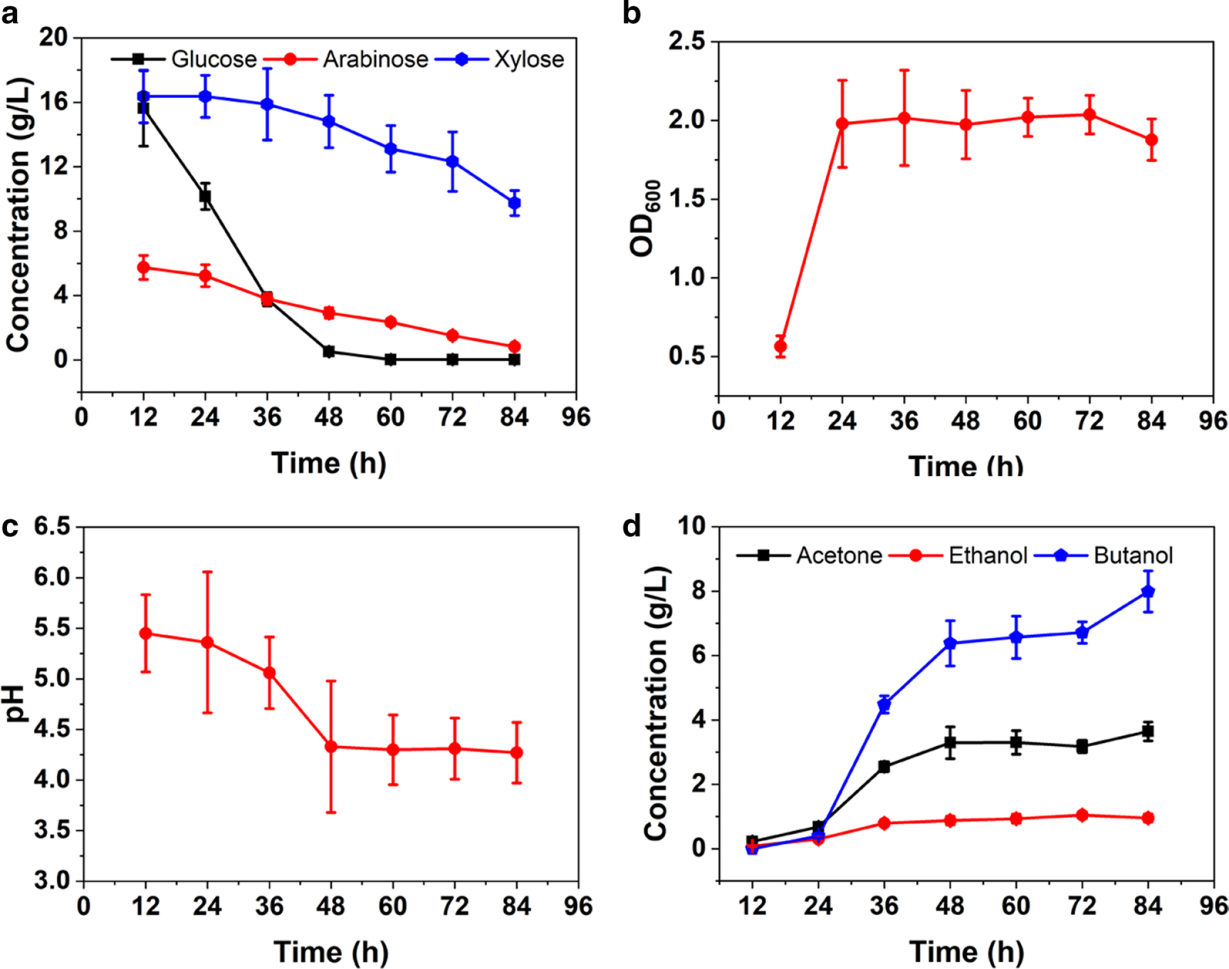
The ABE fermentation process of *C. acetobutylicum* Tust-001. **a** The substrate consumption process; **b** and **c** the changes of OD_600_ and pH during fermentation; **d** the production of solvents during fermentation

As a contrast, the strain with highest butanol yield was named *C. acetobutylicum* Tust-001. The fermentation performance is given in Fig. 7. As shown, *C. acetobutylicum* Tust-001 consumed almost all the glucose (99%) and arabinose (91%), but only 48% of xylose. *C. acetobutylicum* always utilizes the preferred sugars before consuming any other sugars present when grown on a mixture of sugars, as is the case for many bacteria [50]. This process of preferred sugar consumption is known as carbon catabolite repression. Based on our result, the utilization order is glucose > arabinose > xylose, indicating that xylose is not appreciably fermented even in the presence of arabinose. Based on the data of Fig. 7b, c, the first 24 h was the rapid growth phase. 24–48 h was the typical acid production phase, where pH decreased from 5.5 to 4.5 because of acid accumulation. During this phase, strains ceased to grow as OD stayed stable over time. A steady-state solvent production period was obtained between 48 h and 84 h, and the final concentration of acetone, butanol and ethanol reached 3.64 g/L, 7.98 g/L, and 0.95 g/L, respectively. Total solvent production arrived at 12.56 g/L, which were higher than the previous report employing Brewer’s spent grain as substrate [5]. This was accompanied by a butyric acid concentration of approximately 2.18 ± 0.32 g/L and an acetic acid concentration of approximately 1.91 ± 0.07 g/L (data not shown). After the steady-state solvent production phase, the butanol production decreased dramatical as well as the OD_600_ value. This degeneration is attributed to the complex morphological behavior of this strain. In other words, cells entered the spore morphogenesis phase [51]. To the best of our knowledge, this is the first study realizing the direct utilization of VR from solid-state fermentation of cereals and bran for butanol production. Our study has great application potential because the VR is quite easily available and cheap but not well-utilized.

In the future study, many established methods could be employed to further improve our study, which includes (i) metabolic engineering of *C. tyrobutyricum* for n-butanol production through co-utilization of glucose and xylose [52]; (ii) co-culture strategy [53] and (iii) process reinforcement strategy, including gas stripping [54] and new bioreactors [55, 56].

## Conclusions

The biotransformation of VR for butanol was achieved. The dramatic pretreatment effect of SE can be attributed to two aspects: (i) depolymerize the macromolecule glycan into fermentable sugars; (ii) effectively increase the enzymatic digestibility of VR. Inverse strategy employing ARTP and repetitive domestication for strain breeding is quite feasible, providing us with a new tool for solving the problem in the biofuel fields. Our work could be effectively improved by integrating with existing mature enhancement strategies for butanol fermentation.

## Methods

### Microorganism and culture conditions

The working strain *C. acetobutylicum* Tust-001 was derived from *C. acetobutylicum* ATCC 824 by domestication using furfural stress. Strains were maintained in spore suspensions at − 70 °C before experiment. Spores culture were inoculated in clostridial growth medium (**TYA**) containing per liter: 40.0 g glucose, 2.0 g beef extract, 2.0 g yeast extract, 6.0 g tryptone, 3.0 g ammonium acetate, 0.5 g KH_2_PO_4_, 0.5 g K_2_HPO_4_, 0.2 g MgSO_4_·7H_2_O, 0.01 g FeSO_4_·7H_2_O. Prior to cultivation, the cultures were pasteurized for 10 min at 80 °C to inactivate vegetative cells. 10 mL of the resulting seeding suspension was inoculated into 250-mL serum bottle with 100 mL fermentation medium, which was the mixture solvent with enzymatic hydrolysates and the liquid fraction resulting from the SE pretreatment. Before fermentation, the medium was adjusted to pH 5.5 using NaOH 3 M, and then autoclaved at 115 °C for 30 min. After sterilization, a vitamin solution (0.001 g/L PABA, 0.001 g/L thiamine and 0.00001 g/L biotin), a salt solution (0.20 g/L MgSO_4_, 0.01 g/L MnSO_4_, 0.01 g/L FeSO_4_, 0.01 g/L NaCl) and acetate buffer solution (0.50 g/L KH_2_PO_4_, 0.50 g/L K_2_HPO_4_ and 2.20 g/L ammonium acetate) were added to the medium. Procedures requiring strictly anaerobic conditions were done in an anaerobic chamber with 90% N_2_ and 10% H_2_ (GeneScience AG300 Anaero-station, USA).

### Raw material

Fresh industrial vinegar residue was provided by Zilin Vinegar Industry Co., Ltd, a vinegar factory located in Qingxu (Taiyuan, Shanxi, China) and stored at − 20 °C. Before pretreatment, it was dried at 50 °C and sealed in a plastic container.

### Chemicals

Glucose, arabinose, cellobiose, acetic acid, butyric acid, acetone, butanol, ethanol and β-glucosidase were purchased from Solarbio Biological Co., Ltd (Beijing, China). Some other chemicals of analytical grade obtained from Tianjin Fuchen chemical reagent factory (Tianjin, China) were MgSO_4_, MnSO_4_, FeSO_4_, NaCl, KCl, KH_2_PO_4_, K_2_HPO_4_, ammonium acetate, H_2_SO_4_, NaOH, Ca (OH)_2_, and activate carbon. Xylose, d-biotin and l-Cysteine HCl were purchased from Beijing Biotopped Science & Technology Co., Ltd (Beijing, China). The mixed compressed gas (10%H_2_ and 90%N_2_) used to manufacture anaerobic conditions comes from Binhai China (Tianjin, China).

### Steam explosion pretreatment

SE treatment was performed in a 5-L batch vessel (Weifang Derui Biotechnology Co., Ltd., China) which was composed of a reaction retort, a receiving tank and a saturated steam generator with a maximum pressure of 3.5 Mpa. First, the VR was mixed homogeneous with water with the ratio of 7:3. Then, the mixture was top-loaded into the reaction retort and possessed at a certain saturated steam pressure of 1.5 MPa (197 °C), 2 MPa (212 °C) and 2.5 MPa (222.9 °C) until reaching the desired time of 1, 2, 3 and 4 min, respectively. The pretreatment severity was defined as Eq. 3.

3 $$R_{0} = t \times e^{{\frac{T - 100}{14.75}}}$$

where t is the pretreatment time in minutes and T the pretreatment temperature in degrees Celsius [57]. In this paper, $\log (R_{0} )$ was used as pretreatment severity.

After pretreatment, vinegar residue was collected. The treated VR was dried at 50 °C, weighted and mixed with deionized water in a ratio of 1:10 and placed in a water bath of 60 °C for 3 h. After solid–liquid separation, two washes were performed and the resulting water extraction was used to analyze monosaccharide on the surface of treated VR. In addition, the treated-vinegar residue was also mixed directly with a certain volume of deionized water to analyze furfural and HMF. The mass balance of the steam explosion treatment followed the method of Weber et al. [20].

### Enzymatic hydrolysis

The enzymatic hydrolysis was performed in 250-mL flasks in a linear shaking bath using pretreated VR and distilled water. The experimental conditions were adjusted to pH 5.0, 150 rpm, 50 °C, 72 h and a solid load of 5% (w/w). The enzyme load was 15 FPU/g dry matter (DM) of Celluclast 1.5 L and 15 U/g DM of β-glucosidase. In the enzymatic hydrolysis process, the samples were withdrawn every 12 h to analyze monosaccharides and enzymatic hydrolysis rate. Vacuum-filtered hydrolysates were stored at 4 °C until their use for biobutanol fermentation. The monosaccharide recovery rate was calculated following the method of Plaza et al. [5].

### Analytical methods

#### Composition analysis of VR

The VR was characterized using the analytical procedures of the National Renewable Energy Laboratory [58]. The soluble protein was detected using Total protein quantitative test kit produced by Nanjing Jiancheng Bioengineering Institute. The detection was carried out following the kit Manual. The concentrations of sugars, organic acids, ABE solvents and potential inhibitors were determined by HPLC (Aglient1200, USA). The detector was based on the refractive index measurement (Waters 2414 Refractive Index Detector). An Aminex HPX-87H (300 mm × 7.8 mm, Bio-rad, Hercules, CA) column was used, enabling the quantification of glucose, xylose, arabinose, cellobiose, acetic acid, lactic acid, butyric acid, furfural, HMF, ethanol, acetone and butanol. Operational conditions were 0.5 mM H_2_SO_4_ as the mobile phase, at a flow rate of 0.6 mL/min and 30 °C (solvents) and 65 °C (sugars, furfural and HMF). Samples were previously centrifuged at 12,000 rpm over 2 min and filtered through 0.22-µm nylon filters. Hydroxymethyl furfural and furfural were detected using the method of Liu [59].

#### Scanning electron microscopy (SEM) analysis

Pretreated biomass samples were studied by scanning electron microscopy (SEM) using A JEOL JSM–6700F system (JEOL, Japan) to get SEM images (5000× magnification). Before measurement, samples were frozen in liquid nitrogen and dried in a vacuum freeze–dryer. Then, they were coated with a thin layer of gold using a sputter-coater (Hitachi Science Systems, Tokyo, Japan).

#### Pore and specific surface area analysis

The porous property characterization was measured by nitrogen adsorption and desorption isotherms on a Beshide 3H-2000PS2 sorption analyzer (BeiShiDe Instrument, China) followed the method of Su W [33]. The samples were degassed in a vacuum at 353 K for 6 h before measurement. The specific surface area in the relative pressure range between 0.04 and 0.16 was calculated by the multipoint BET method. The total pore volume at a relative pressure of 0.99 was estimated according to the BJH method. Pore size distribution was calculated according to the BJH method from the desorbed amount of liquid nitrogen.

### ARTP mutation method

The mutation was carried out on ARTP-IIIS made by Wuxi TMAXTREE Biotechnology Co., Ltd, Wuxi, China. The instrument consists of a radiofrequency (13–56 MHz) power supply, a co-axial type plasma generator, a gas supply control subsystem, and a stainless-steel plate. The plate can be moved up and down to adjust the distance between the plasma torch nozzle exit and the treated sample. During the mutant, pure helium was used as the plasma working gas containing different chemically active species, which can be irradiated upon the sample on the stainless-steel plate at the downstream of the plasma torch nozzle exit for microbial mutation [60]. The radiofrequency power input was set at 120 W and the distance between the plasma torch nozzle exit and the sample plate (D) was fixed at 2 mm. Under these fixed conditions, the mutagenesis dosage by ARTP was dependent on the treatment period [60]. For the mutagenesis, strains were harvested during the log-phase and 10 μL of cell suspension (10^7^ to 10^8^ cells per milliliter) was spread on the sterilized steel plate and exposed to the ARTP system’s nozzle exit for 0, 30, 60, 90, 120, 150 and 180 s. After each treatment, the samples were all eluted with 1 mL sterile water into a new tube, properly diluted, and then grown on a solid medium for 48 h at 37 °C prior to determine the lethality rate. The individual colonies on the control medium and each mutated medium were counted. The lethality rate was determined as follows:

4 $$Lethality\;rate(\% )\; = \frac{control\;colonies - survival\;colonies}{control\;colonies}\; \times \;100\% .$$

### Repetitive domestication

First, 0.5 mL ARTP-treated cells of ancestral log-phase culture were transferred into TYA tubes in presence of 0.5 g/L furfural. After 2-day culture at 37 °C, cells which could grow in the tubes were centrifuged and spread onto TYA agar plates with a higher furfural concentration and cultivated at 37 °C for 2 days, and then colonies were selected to continue the next experimental cycle. The furfural concentration gradient was set at from 0.5, 0.7, 0.9…to 4 g/L. If being inhibited, the cells from the previous cycle were mutated by ARTP and the domestication experiment was continued.

To prevent cells from going extinct or being completely inhibited before any resistance mutants occurred, cell from each experimental line should be backed up. In sequence, as the experimental cycle was carried forward, the furfural concentration was increasing and furfural tolerance of cells would be also enhanced throughout this experimental domestication.

### Exploration of the mutagenized strain’s genetic stability

Genetic stability was carried out followed the method proposed by Zhang et al. [60]. In brief, the mutant strain was cultivated on solid medium for approximately 48 h. Then, single colonies were selected and streaked onto a new plate for another 48 h of cultivation. This experimental procedure was performed repeatedly for total of 20 subcultures in anaerobic incubator. Finally, the inhibitory tolerance of each subculture was evaluated by the fermentation procedure described in “Microorganism and culture conditions” section.

## Supplementary information

**Additional file 1.** The detailed data analysis procedure of spearman correlation, univariate and multivariable *GAM.*

**Additional file 2.** The detailed model derivation process for enzymolysis kinetic equation.

**Additional file 3.** The concrete process of the model fitting and the result evaluation for Eq. 2.

**Additional file 4.** The N2 adsorption in data in detailed.

## Data Availability

The datasets supporting the conclusions of this article are included within the article

## Abbreviations

SE: Steam explosion
VR: Vinegar residue
MPa: Megapascal
DM: Dry matter
ARTP: Room temperature plasma
GAM: Generalized additive model
