# Supplementary material for: Development of optimal steam explosion pretreatment and highly effective cell factory for bioconversion of grain vinegar residue to butanol
Source: Biotechnol Biofuels. 2020 Jun 24;13:111. doi: 10.1186/s13068-020-01751-7 (PMC7315531; doi:10.1186/s13068-020-01751-7)
Supplement: Supplementary file 1 — Additional file 1. The detailed data analysis procedure of spearman correlation, univariate and multivariable GAM. [file 13068_2020_1751_MOESM1_ESM.docx]

**Additional file 1: The setup of generalized additive model (GAM)**

**1. The spearman correlation analysis among the operation parameters and sugar yield**

The spearman correlation coefficient was calculated with R, and the results were given in S-Table 1.1.

S-Table 1.1 The spearman correlation coefficient matrix calculated using Spearman's correlation method

|  | ***Pressure*** | ***Holding Time*** | ***Severity*** | ***Glucose*** | ***Xylose*** | ***Arabinose*** |
| --- | --- | --- | --- | --- | --- | --- |
| ***Pressure*** | 1 | 0 | 0.8278 | 0.473 | 0.4139 | 0.473 |
| ***Holding Time*** | 0 | 1 | 0.5398 | 0.8205 | 0.8853 | 0.8205 |
| ***Severity*** | 0.8278 | 0.5398 | 1 | 0.8601 | 0.8322 | 0.8462 |
| ***Glucose*** | 0.473 | 0.8205 | 0.8601 | 1 | 0.958 | 0.958 |
| ***Xylose*** | 0.4139 | 0.8853 | 0.8322 | 0.958 | 1 | 0.9091 |
| ***Arabinose*** | 0.473 | 0.8205 | 0.8462 | 0.958 | 0.9091 | 1 |

S-Table 1.2 The matrix of *p*-values for testing the hypothesis of no linear correlation against the alternative hypothesis of a nonzero correlation

|  | ***Pressure*** | ***Holding Time*** | ***Severity*** | ***Glucose*** | ***Xylose*** | ***Arabinose*** |
| --- | --- | --- | --- | --- | --- | --- |
| ***Pressure*** | 1 | 1 | 0.000885 | 0.120373 | 0.181015 | 0.120373 |
| ***Holding Time*** | 1 | 1 | 0.070065 | 0.001077 | 0.000129 | 0.001077 |
| ***Severity*** | 0.000885 | 0.070065 | 1 | 0.000332 | 0.000785 | 0.000521 |
| ***Glucose*** | 0.120373 | 0.001077 | 0.000332 | 1 | 9.54E-07 | 9.54E-07 |
| ***Xylose*** | 0.181015 | 0.000129 | 0.000785 | 9.54E-07 | 1 | 4.19E-05 |
| ***Arabinose*** | 0.120373 | 0.001077 | 0.000521 | 9.54E-07 | 4.19E-05 | 1 |

Obviously, the correlation between the operation parameters and glucose recovery followed the order: Severity> Holding time > Pressure based on the correlation coefficient, which was consistent with the results of two factors analysis of variance (ANOVA) in the manuscript. **In the following part, the statistical relationships between parameters of the steam explosion treatment and sugar recovery will be fully exploited using GAM analysis.**

**2. The setup of** ***Generalized Additive Models* *(GAM)* for sugar recovery prediction**

In order to better understand the nonlinear phenomenon among SE operation parameters and sugar recovery, the analysis of the generalized additive models was performed using ***pyGAM***^[1]^.

**2.1 The strategy for optimization of GAM**

The Generalized additive models, in general can be described as following:

$$\text{y}\sim E\text{xponentialFamily(}\mu|X)$$

Where

$$g(\mu|X)=\beta_{o}+f_{1}(X_{1})+f_{2}(X_{2},X_{3})+...+f_{M}(X_{N})$$

Obviously, a GAM has 3 components. According to the document of pyGam (https://pygam.readthedocs.io/en/latest/index.html), each component can be optimized by setting the different parameters, which was summarized as follow:

- *Distribution*, which including *‘normal’*,*’binomial’*,*’possion’*, *‘gamma’* and *‘inv_gauss’*;
- *Link function: Link functions take the distribution mean to the linear prediction. So far, the following are available. ‘identity’,’logit’,’inverse’,’log’,’inverse-squared’.*
- *Fucntional Form:* *linear* terms, *spline* terms, *factor* terms, *tensor* productions and *intercept*.

The optimization procedure for the GAM models was shown in Figure S-1.1.


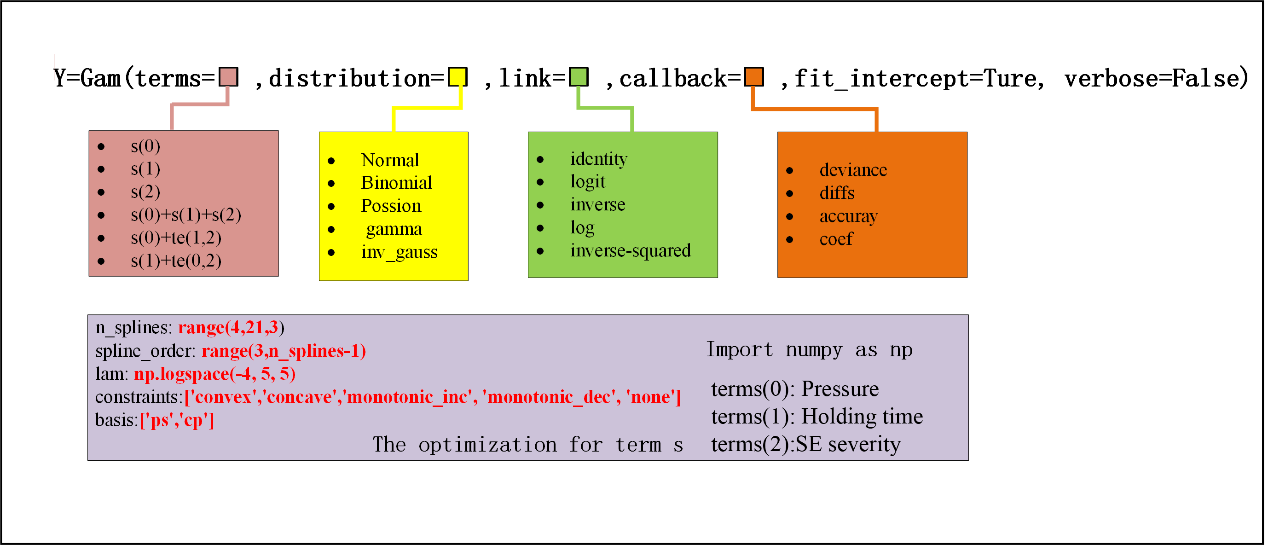


Figure S-1.1 The optimization procedure of our GAM model. Five main items were optimized including *terms*, *distribution*, *link function*, *callback,* and Spline term with the function ***gam*** in ***pygam*** package. The optimization method for each item was show in the box in details.

In general, Lam, short for λ, controls the strength of the regularization penalty on each term. the generalized additive models was determined by Generalized Cross Validation (GCV) minimization and R2 (explained deviance) maximization [2].

**2.2 The core codes for the setup of GAM**

The example takes the ***LinearGAM*** Model as example. The explanation for each parameter is listed as following: The other four type of GAM were set up using the same method.

**spline:** Number of splines to use for the feature function.

**constraints:** Type of constraint to apply to the term.

**lam:** Strength of smoothing penalty

**link function**: the defaulted ***Identity***

| # Python language  Spline = range(4, 21, 3)   #  constraints = ['convex', 'concave', 'monotonic_inc', 'monotonic_dec', 'none']  lam = np.logspace(-4, 5, 5)  lams = [lam] * 3    **for** i0 in Spline:  **for** p0 in constraints:  **for** i1 in Spline:  **for** p1 in constraints:  **for** i2 in Spline:  **for** p2 in constraints:                              gam = LinearGAM(s(0, n_splines=pars[0], constraints=pars[1])                                              + s(1, n_splines=pars[2], constraints=pars[3])                                              + s(2, n_splines=pars[4], constraints=pars[5]))                              gam.gridsearch(PTS, A, lam=lams)  gcv= gam.statistics_['GCV']  R2= gam.statistics_['pseudo_r2']['explained_deviance'] |
| --- |

**2.3 The** **optimized results of the GAM models**

Taking the ***LinearGAM*** Model as example, there are 900 models generated, whose GCV and R2 were shown as Figure S-2. X axis represents the 900 models. The models were ordered by the their GCV values, which were displayed by red line (the left Y axis). The corresponding R2 (explained deviance) of the models were displayed by black dots (the right y axis). As Figure S-1.2 shown, the miner GCV does not always have higher explained deviance. Therefore, the ideal model must exhibit both minor GCV and high R2.


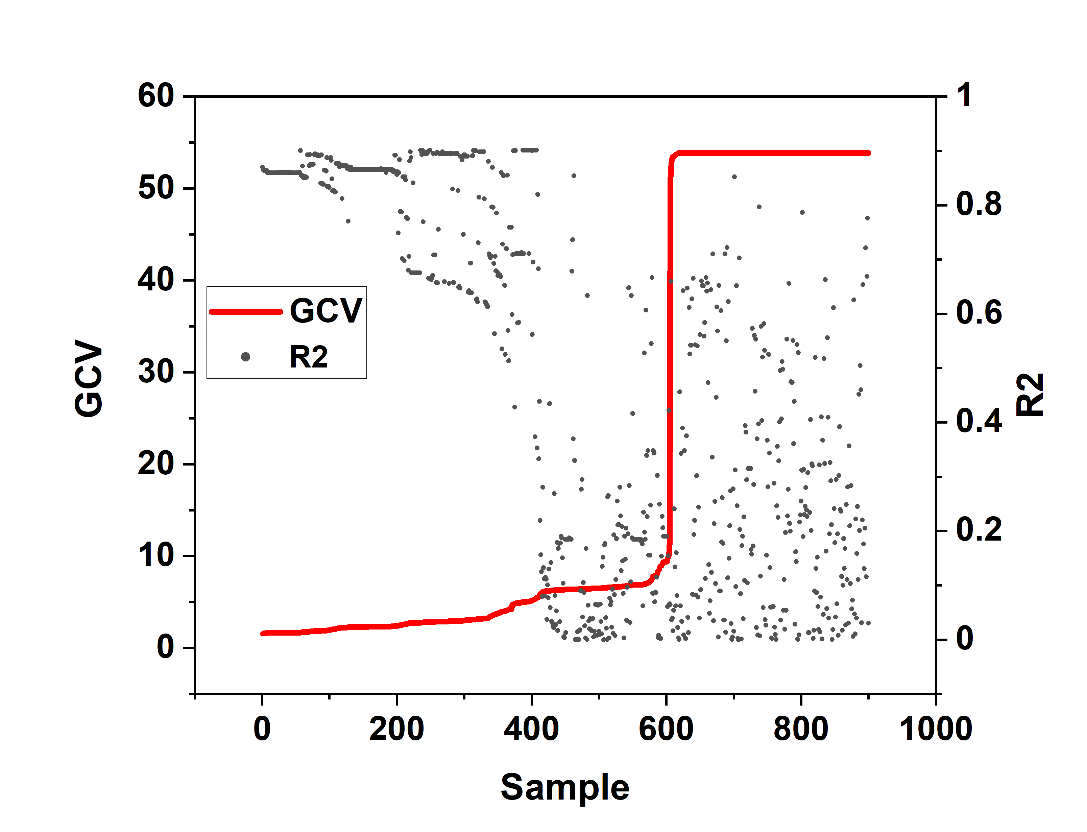


Figure S-1.2 GCV and R2 of the models generated by the LinearGAM methods. The models were ordered by the their GCV values, which were displayed by red line (the left Y axis). The corresponding R2 (explained deviance) of the models were displayed by black dots (the right y axis).

***Based on the optimized methods, we found that LinearGAM model perfomed better than the other four method (Briefly, the GCV and R2 of the most LinearGAM models were better).*** Because each type of GAM generated ***more than 900 models***, the data was not shown. *Readers can run the core python code to get the results.* Figure S-3 showed the final candidate models obtained from ***LinearGAM*** with different functional forms.


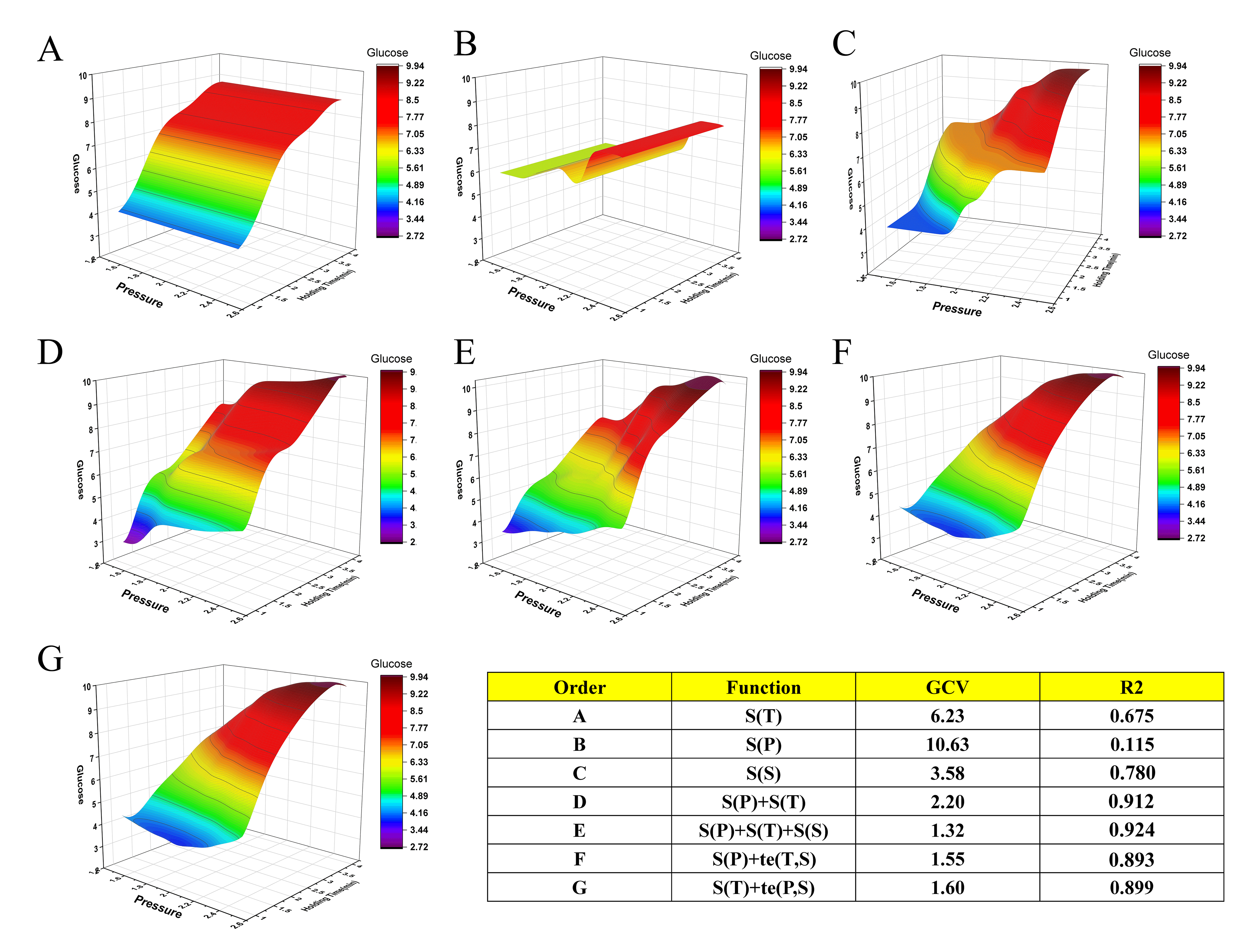


Figure S-1.3 The candidate models generated with different function terms. Each of them was selected by comparing R2 and GCV from more than 900 models respectively with the same function term. In A-G, X-axis represents pressure (mPa), Y-axis represents holding time (min), Z-axis represents monosaccharide recovery (Glucose g/100g). S (outside the bracket) represents spline term; te represents tensor term. P represents pressure, H represents holding time, S (inside the bracket) represents the severity.

As Figure S-1.3 shown, models A,B,C are univariate GAM. The AIC (Akaike’s Information Criteria) value of pressure, holding time and severity were 63.6, 52.16 and 47.87 respectively. The GCV and R2 of the corresponding models were (6.23, 0.675), (10.63, 0.115) and (3.58,0.78) respectively. **The lower AIC and GCV represent higher interpretation degree. Therefore, the correlation between the operation parameters and glucose recovery followed the order: Severity> Holding time > Pressure**[3]**.**

**2.4 The validation of the candidate models**

To validate the candidate models, additional experiments were carried out. The R2 between the experiment results and model prediction were analyzed. The higher R2 score represents the higher accuracy of the model^[1]^. The highest one will be the best GAM model in this study. The experiment data was given in **S-Table 1.3 at the end of this supplementary**. And the prediction performance of the candidate models was shown in S-Figure 1.4


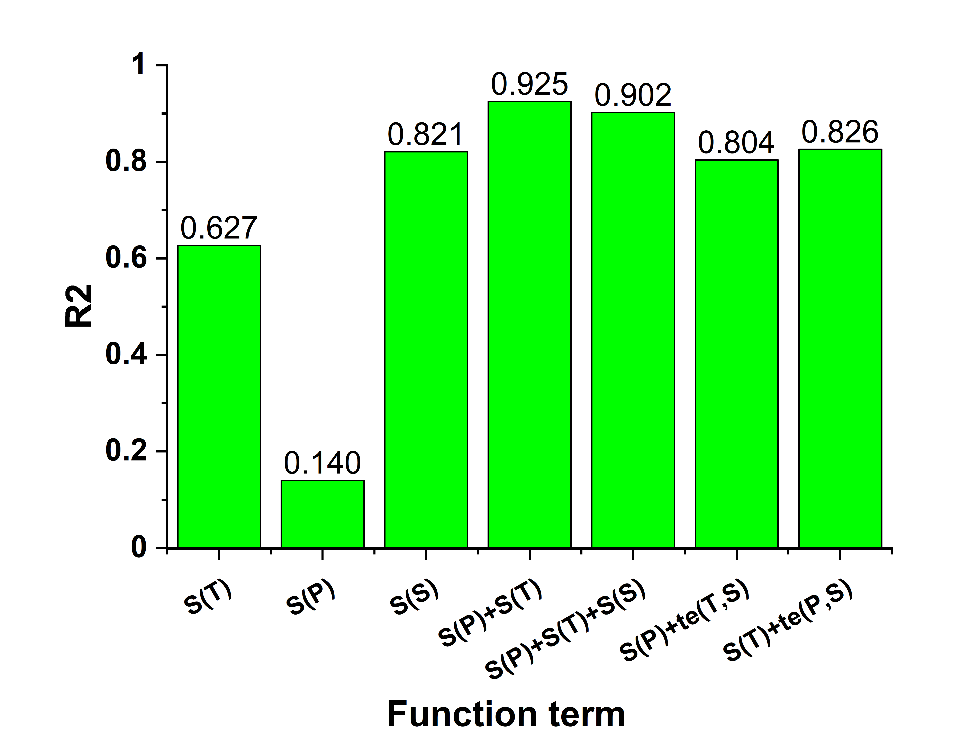


Figure S-1.4 The prediction result of the indicate models for the addition experiment. S (outside the bracket) represents spline term; te represents tensor term. P presents pressure, H presents holding time, S (inside the bracket) represents the severity.

The prediction result showed that the model with function term S(P)+S(T) provided the best result with a R2 as high as 0.925. Therefore, it was chosen as the final model to describe the complex nonlinear relationship between operation condition and sugar recovery.

The setup of GAM model for xylose and arabinose follows the same method. Therefore, the detailed procedure was shown in this supplementary material and the key result from model setup and prediction using the additional experiment data was shown in S-Table S-1.4

Table S-1.4 the key result from model setup and prediction using the additional experiment data

| Sugar | Result from model setup | | Result for prediction |
| --- | --- | --- | --- |
|  | GCV | R2 | R2 |
| Xylose | 1.26 | 0.924 | 0.905 |
| Arabinose | 1.18 | 0.936 | 0.916 |

3. The comparison of GAM and 3D Interpolation method

To In order to better understand the above nonlinear phenomenon, we also introduced the 3D colormap surface to visualize all the experiment data, which took the pressure as the x axis, the residence time as the y axis, and the yield of simple sugar as the z axis. The key code of Matlab version for this method was shown as following:

| %Matlab code  % data was a matrix with 3 columns, which represent pressure, holding time and sugar yield respectively.  % the document for each Matlab function can be found at: <https://ww2.mathworks.cn/help/documentation-center.html>  *x =data(:,1);*  *y = data(:,2);*  *z = data(:,3);*  *number=500; % number was the number of points to be generated*  *xlin = linspace(min(x),max(x),number);*  *ylin = linspace(min(y),max(y),number);*  *[X,Y] = meshgrid(xlin,ylin);*  *Z = griddata(x,y,z,X,Y,'cubic');*  *surf(X,Y,Z); shading interp;* |
| --- |

Table S-1.5 the key result from model setup and prediction using the additional experiment data using 3D Interpolation

| Sugar | R2from model setup | Result for prediction |
| --- | --- | --- |
|  | R2 | R2 |
| Glucose | 1.00 | 0.832 |
| Xylose | 1.00 | 0.852 |
| Arabinose | 1.00 | 0.814 |


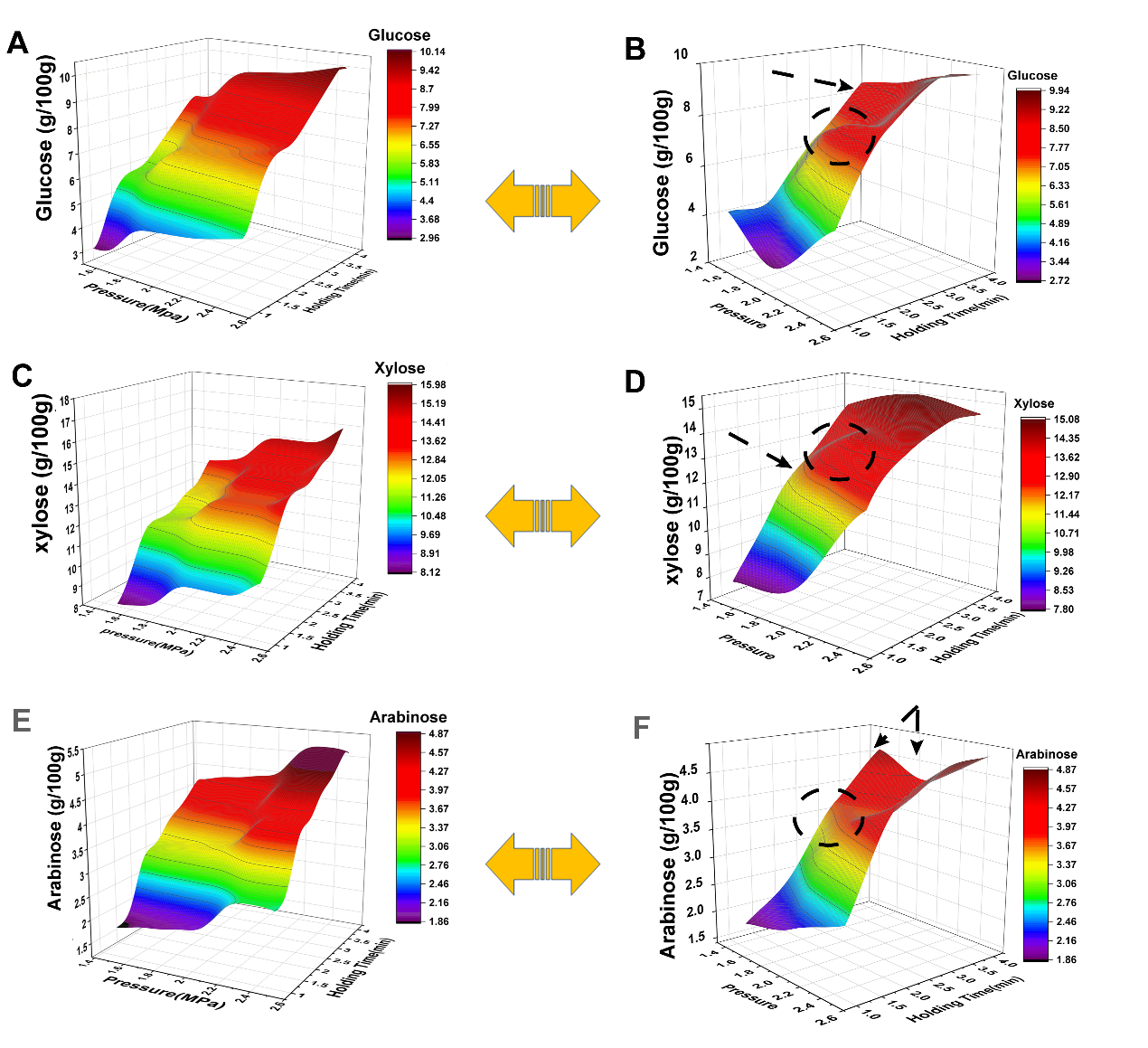


Figure S-1.5 the comparison of GAM model and 3D Interpolation.

As Table S-1.5 and Figure S-1.5shown, the fitting performance of 3D Interpolation can reach 100%, but the prediction accurate was lower that of GAM. Obviously, there exists overfitting phenomenon in the 3D Interpolation method. Figure S-1.5 showed the overfitting with black dash line. **It can be concluded that GAM works better on exploring the complex nonlinear relation in our study.**

S-Table 1.3 the additional experiment result for the model validation

| Pretreatment conditions | | |  | Composition of the washing liquid (g/100g feedstock) | | |
| --- | --- | --- | --- | --- | --- | --- |
| Pressure（MPa） | Time（min） | Severity  (Log(R_0_)) |  | Glucose | Xylose | Arabinose |
| 1.75 | 1 | 3.09 |  | 3.60±0.22 | 7.30±1.10 | 1.76±0.12 |
| 1.75 | 2 | 3.39 |  | 5.61±0.53 | 12.71±1.08 | 3.38±0.47 |
| 1.75 | 3 | 3.56 |  | 6.53±0.36 | 14.19±0.80 | 4.18±0.57 |
| 1.75 | 4 | 3.69 |  | 8.13±1.22 | 16.81±1.92 | 3.97±0.48 |
| 2.25 | 1 | 3.47 |  | 3.46±0.60 | 9.20±0.93 | 2.28±0.12 |
| 2.25 | 2 | 3.77 |  | 7.25±0.80 | 12.35±0.97 | 3.68±0.28 |
| 2.25 | 3 | 3.95 |  | 7.37±0.87 | 15.32±1.30 | 4.49±0.34 |
| 2.25 | 4 | 4.07 |  | 8.81±0.76 | 17.02±0.90 | 4.22±0.63 |

**Reference**

1. Servén D, Brummitt C. pyGAM: generalized additive models in python. March; 2018;

2. Gómez Rubio V. Book Review: Generalized Additive Models: An Introduction with R (2nd Edition). Journal of statistical software. 2018;86.

3. Liu L, Dong Y, Kong M, Zhou J, Zhao H, Wang Y, et al. Towards the comprehensive water quality control in Lake Taihu: Correlating chlorphyll a and water quality parameters with generalized additive model. Science of The Total Environment. Elsevier; 2020;705:135993.
