# Supplementary material for: Development of optimal steam explosion pretreatment and highly effective cell factory for bioconversion of grain vinegar residue to butanol
Source: Biotechnol Biofuels. 2020 Jun 24;13:111. doi: 10.1186/s13068-020-01751-7 (PMC7315531; doi:10.1186/s13068-020-01751-7)
Supplement: Supplementary file 2 — Additional file 2. The detailed model derivation process for enzymolysis kinetic equation. [file 13068_2020_1751_MOESM2_ESM.docx]

**Additional file 2**

Cellulose + water → glucose

According to chemical reaction kinetics：

r——Enzymatic hydrolysis reaction rate，*g﹒(L﹒h)^-1^*；

C——Cellulose concentration，*g/L*；

t——Enzymatic hydrolysis residence time，*min*；

n——Enzymatic hydrolysis reaction order；

k——Enzymatic hydrolysis rate constant，*(g/L)^-(n-1)^/h*

Then Integrate it, when n=1:

When n>1:

$$C=\frac{1}{\left(-\left(n-1\right) \left(C_{0}-k t\right)\right)^{\frac{1}{n-1}}}$$

Substitute the enzymolysis data (x: Cellulose concentration, t- Enzymatic hydrolysis time (h)) into above formula. The correlationship between the real Cellulose concentration the predicted concentration R^2^ was used to evaluate the model accuracy.





Based on the R2 analysis, n was 2 in our study.
