# Supplementary material for: Development of optimal steam explosion pretreatment and highly effective cell factory for bioconversion of grain vinegar residue to butanol
Source: Biotechnol Biofuels. 2020 Jun 24;13:111. doi: 10.1186/s13068-020-01751-7 (PMC7315531; doi:10.1186/s13068-020-01751-7)
Supplement: Supplementary file 3 — Additional file 3. The concrete process of the model fitting and the result evaluation for Eq. 2. [file 13068_2020_1751_MOESM3_ESM.docx]

**Additional file 3**

**2-a The Model fitting process**

**
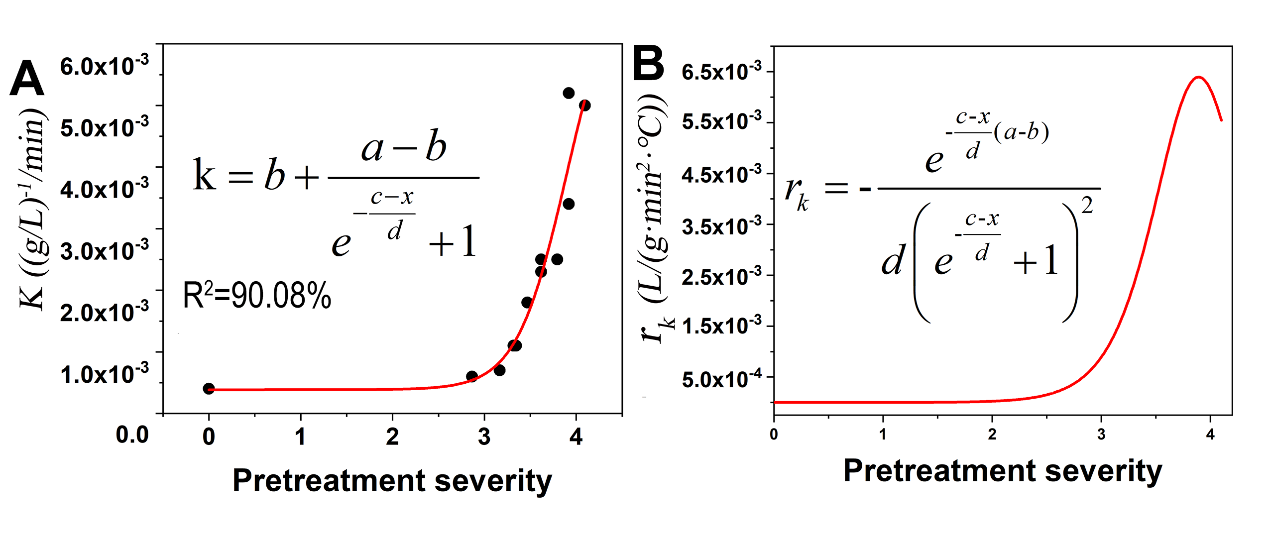
**

The fitting code and explanation running in Matlab software (The MathWorks, Inc).

1. ***beta0=[3.86208E-4 0.00734 3.89205 0.2718] %* Initial value of parameters a,b,c,d**
2. ***F=@(A,x)((A(1)-A(2))./(1+exp((x-A(3))./A(4)))+A(2)) % The formula***
3. ***beta = nlinfit(X,Y,F,beta0) %obtained the values of a,b,c,d***
4. ***syms x a b c d***
5. ***A(1)=a***
6. ***A(2)=b***
7. ***A(3)=c***
8. ***A(4)=d***
9. ***F1=F(A,x)***
10. ***f=diff(F1,x) % the formula of r_k_***

**2-b Evaluation of model**

**Table S3-1 The values of constants (a, b, c, d) involved in the equation 4**

| **Constants** | **Value** | **Standard Error** | **t-Value** | **Prob>｜t｜** | **Dependency** |
| --- | --- | --- | --- | --- | --- |
| a | 3.86208E-4 | 4.55217E-4 | 0.8484 | 0.42087 | 0.791 |
| b | 0.00734 | 0.00432 | 1.69973 | 0.1276 | 0.98813 |
| c | 3.89205 | 0.34945 | 11.13758 | 3.77683E-6 | 0.98912 |
| d | 0.2718 | 0.14921 | 1.82159 | 0.10599 | 0.92545 |
