# Supplementary material for: Development of optimal steam explosion pretreatment and highly effective cell factory for bioconversion of grain vinegar residue to butanol
Source: Biotechnol Biofuels. 2020 Jun 24;13:111. doi: 10.1186/s13068-020-01751-7 (PMC7315531; doi:10.1186/s13068-020-01751-7)
Supplement: Supplementary file 4 — Additional file 4. The N2 adsorption in data in detailed. [file 13068_2020_1751_MOESM4_ESM.docx]

**Additional file 4**


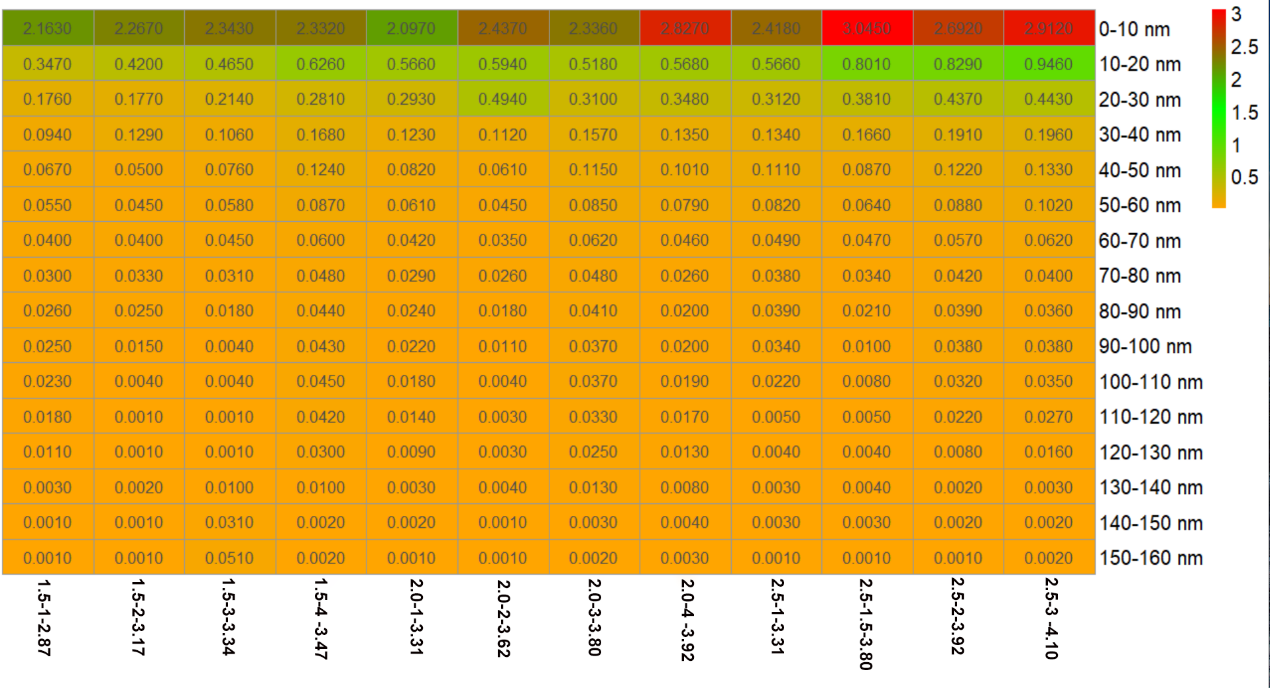


Fig. SD.1. the pore distributions data under different pretreatment conditions. **Each xlabel** represents the pressure-resident time-the corresponding pretreatment severity. **Each ylabel** represents the different pore diameter intervals, and **each Number in the colored table** represents the cumulative hole area of a pore diameter interval. Each column represents one group. Taking the first column (1.5-1-2.87) as example, it represents the sample with steam explosion pretreated at: pressure 1.5, resident time 1min, and the pretreatment severity is 2.87.
